# Baicalin Targets HSP70/90 to Regulate PKR/PI3K/AKT/eNOS Signaling Pathways

**DOI:** 10.3390/molecules27041432

**Published:** 2022-02-21

**Authors:** Yinzhu Hou, Zuqing Liang, Luyu Qi, Chao Tang, Xingkai Liu, Jilin Tang, Yao Zhao, Yanyan Zhang, Tiantian Fang, Qun Luo, Shijun Wang, Fuyi Wang

**Affiliations:** 1 Beijing National Laboratory for Molecular Sciences, CAS Research/Education Center for Excellence in Molecular Sciences, CAS Key Laboratory of Analytical Chemistry for Living Biosystems, National Centre for Mass Spectrometry in Beijing, Institute of Chemistry, Chinese Academy of Sciences, Beijing 100190, China; yzhhou1995@iccas.ac.cn (Y.H.); zuqliang@iccas.ac.cn (Z.L.); qiluyu@iccas.ac.cn (L.Q.); 15110098635@163.com (C.T.); liuxingkai@iccas.ac.cn (X.L.); raytjl@iccas.ac.cn (J.T.); yaozhao@iccas.ac.cn (Y.Z.); zhangyy0816@iccas.ac.cn (Y.Z.); fangtt@iccas.ac.cn (T.F.); 2 College of Chemical Science, University of Chinese Academy of Sciences, Beijing 100049, China; 3 College of Traditional Chinese Medicine, Shandong University of Traditional Chinese Medicine, Jinan 250355, China

**Keywords:** baicalin, chemical proteomics, mass spectrometry, target protein, heat shock protein, PKR signaling, PI3K/AKT signaling, eNOS signaling

## Abstract

Baicalin is a major active ingredient of traditional Chinese medicine *Scutellaria baicalensis*, and has been shown to have antiviral, anti-inflammatory, and antitumor activities. However, the protein targets of baicalin have remained unclear. Herein, a chemical proteomics strategy was developed by combining baicalin-functionalized magnetic nanoparticles (BCL-N_3_@MNPs) and quantitative mass spectrometry to identify the target proteins of baicalin. Bioinformatics analysis with the use of Gene Ontology, STRING and Ingenuity Pathway Analysis, was performed to annotate the biological functions and the associated signaling pathways of the baicalin targeting proteins. Fourteen proteins in human embryonic kidney cells were identified to interact with baicalin with various binding affinities. Bioinformatics analysis revealed these proteins are mainly ATP-binding and/or ATPase activity proteins, such as CKB, HSP86, HSP70-1, HSP90, ATPSF1β and ACTG1, and highly associated with the regulation of the role of PKR in interferon induction and the antiviral response signaling pathway (*P* = 10^−6^), PI3K/AKT signaling pathway (*P* = 10^−5^) and eNOS signaling pathway (*P* = 10^−4^). The results show that baicalin exerts multiply pharmacological functions, such as antiviral, anti-inflammatory, antitumor, and antioxidant functions, through regulating the PKR and PI3K/AKT/eNOS signaling pathways by targeting ATP-binding and ATPase activity proteins. These findings provide a fundamental insight into further studies on the mechanism of action of baicalin.

## 1. Introduction

Baicalin, a flavonoid compound, is one of the major active ingredients of *Scutellaria baicalensis* [1,2], and is also an important component of many traditional Chinese medicine formulae. Pharmacological studies on baicalin have demonstrated that baicalin has a variety of biological activities, e.g., antioxidant and anti-inflammatory [3,4,5], anti-influenza [6], antibacterial [7], antiviral [8], antipyretic [9], and anticancer [2,10]. A recent study revealed that baicalin could significantly activate the activity of CPT1A to accelerate the process of fatty acid degradation [11]. Most recently, baicalin was shown to significantly inhibit the interaction of the spike proteins of SARS-CoV-2 with angiotensin-converting enzyme 2 (ACE2) [12], and to have the potential to fight the cytokine storm in SARS-CoV-2 [1,13]. It has also been reported that baicalin could suppress the progression of Type 2 diabetes-induced liver tumors by regulating the METTL3/m6A/HKDC1 axis and downstream Caspase3 pathway [14], and retard neuron autophagy and apoptosis via the regulation of astrocyte polarization in pentylenetetrazol-induced epileptic rats [15]. These studies were focused on elucidating the mechanism of action of baicalin via the characterization of biological phenotypes [16,17,18], however, the proteome-wide targets of baicalin are still unclear.

The identification of the target proteins of a drug is a key scientific issue in drug discovery [19,20,21,22,23], for which chemical proteomics is the most widely used approach [24,25,26,27]. There are two commonly used strategies for chemical proteomics identification of target proteins of drugs, i.e., activity-based protein profiling (ABPP) [28] and compound-centric chemical proteomics (CCCP) [26,29].

CCCP strategy includes two steps: separation and enrichment of target proteins, and protein identification. During the past decades, photocatalytic affinity microspheres [30], agarose affinity microspheres [31] and the fishing-rod strategy [21,32] have been developed and successively applied for the separation and enrichment of target proteins of small molecular drugs. The core of such affinity separation techniques is fixing the drug molecules or derivatives to a solid matrix by covalent binding [33]. It is crucial that modifications to drug molecules should not change their biological activity [21,34]. Since functionalized nanomaterials were used to selectively enrich biomolecules in the 1970s [35], they have attracted increasing attention in the isolation and enrichment of low abundant proteins or specifically functional proteins for targeted proteomics. A number of assembled or surface-functionalized micro-/nanomaterials are currently used in the enrichment of specific functional proteins in complicated biological samples for precise proteomics analysis [36,37,38]. Our group have previously assembled gold nanoparticles (AuNPs) functionalized with 1,3-*trans*-thiazoplatin (*trans*-PtTz) crosslinked DNA duplexes [32], and magnetic nanoparticles (MNPs) covalently modified with 1,2-cisplatin crosslinked DNA duplexes [39] to enrich cellular proteins which recognize/interact with platinum-damaged DNA. The integration of the functionalized nanoparticles and quantitative mass spectrometry (qMS) analysis allowed us to discover that the nuclear positive cofactor 4 (PC4) recognized and specifically bound to the *trans*-PtTz-crosslinked DNA, and that a few RNA binding proteins interacted with the 1,2-cisplatin-crosslinked DNA with high affinity [32,39].

In recent years, thanks to the development and maturity of chemical proteomics, the specific targets of a number of classic active ingredients of TCMs [40,41,42,43,44], e.g., arsenic trioxide [44], artemisinin [41], have been identified. Besides, by using a biotin modified chemical probe, researchers found that berberine can directly interact with actin to affect actin assembly [34]. Another study, using the photo-affinity labeling approach, revealed that annexin A2 was a direct-binding target of matrine in cancer cells [45].

**Figure 1:**
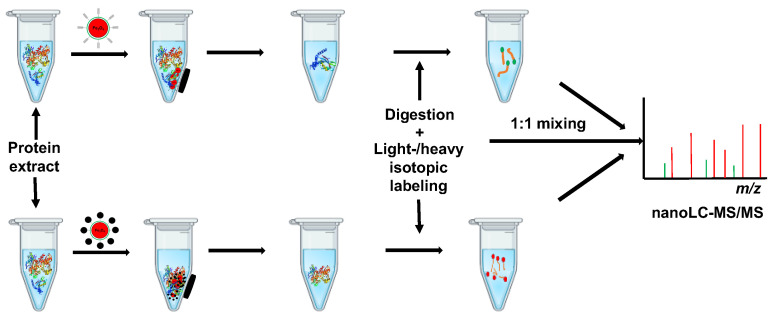
Diagrammatic illustration of the workflow for capture and identification of the target proteins of baicalin (BCL) by using quantitative mass spectrometry following the isolation and enrichment of the target proteins by the BCL-functionalized magnetic nanoparticles.

In the present work, we chemically modified baicalin with a PEG-linked azide group, and then loaded the chemically engineered baicalin on alkynyl magnetic nanoparticles via click reaction to assemble baicalin functionalized magnetic nanoparticles (MNPs) for capturing and enriching the protein targets of baicalin from human embryonic kidney HEK293 cells (Figure 1). The subsequent qMS analysis identified 14 proteins interacting with baicalin. Bioinformatics analysis revealed that the target proteins are mainly associated with the regulation of the role of PKR in interferon induction and the antiviral response signaling pathway, PI3K/AKT signaling pathway and eNOS signaling pathway.

## 2. Results and Discussion

### 2.1. Assembling and Characterization of Baicalin Functionalized Magnetic Nanoparticles

**Figure 2:**
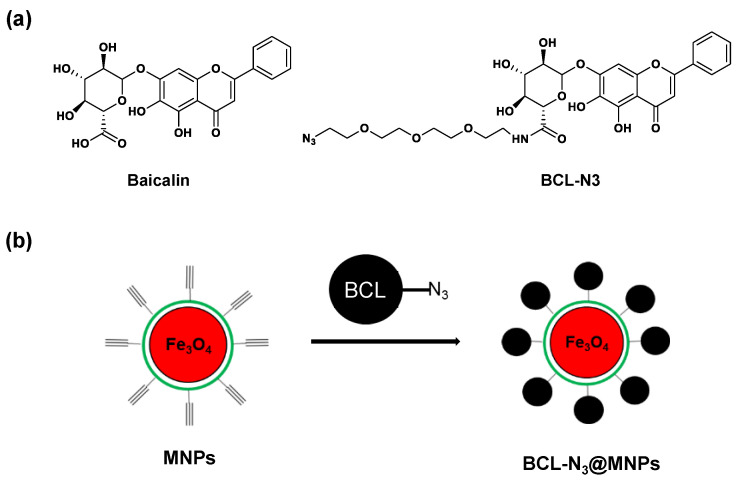
Assembling of baicalin functionalized magnetic nanoparticles (MNPs). (**a**) Chemical structure of baicalin and BCL-N_3_; (**b**) Schematics of magnetic nanoparticles modified with BCL-N_3_.

**Figure 3:**
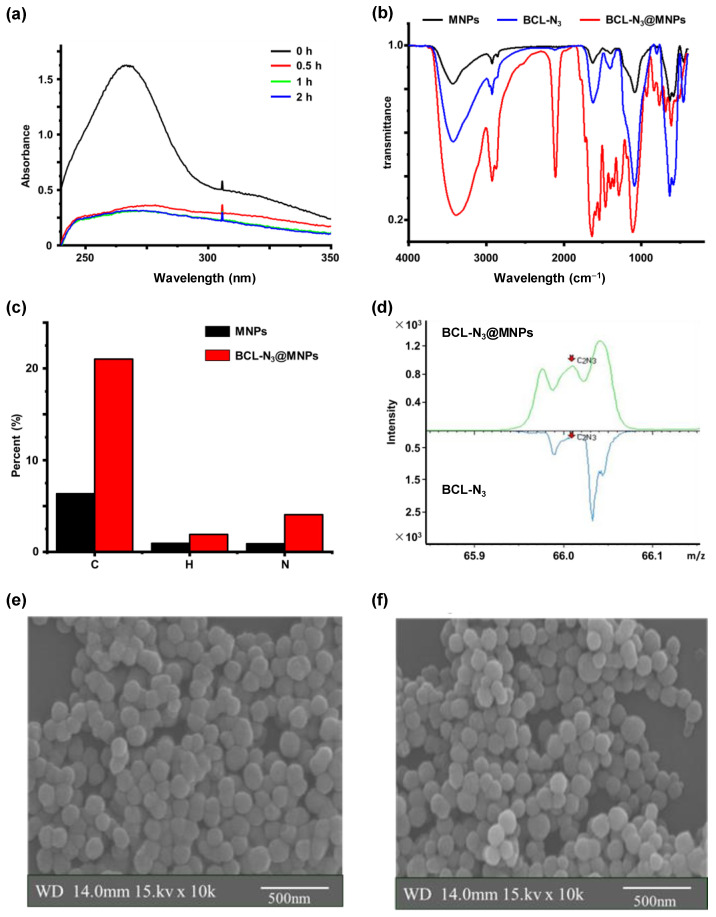
Characterization of BCL-N_3_@MNPs. (**a**) UV spectra of BCL-N_3_ in the supernatant of the reaction mixture of BCL-N_3_ with MNPs at various times; (**b**) IR spectra of BCL-N_3_, intact MNPs and BCL-N_3_@MNPs; (**c**) the percentage of different elements (C, H, N) in MNPs and BCL-N_3_@MNPs; (**d**) enlarged view of ToF-SIMS spectra of MNPs and BCL-N_3_@MNPs; (**e**,**f**) the SEM images of MNPs (**e**) and BCL-N_3_@MNPs (**f**).

To identify the protein targets of baicalin, we first designed and synthesized a baicalin derivative with an azide group attached to the sugaring carboxyl of the baicalin via a PEG chain, while retaining the active skeleton of baicalin, i.e., the structure of flavones. The azido baicalin, designated as BCL-N_3_ (Figure 2a), was characterized by ESI-MS, ^1^H-NMR and ^13^C-NMR (Figure S1–S3 in the Supplementary Materials). BCL-N_3_ was then conjugated with alkynyl ferromagnetic oxide nanoparticles via azido-alkynyl click reaction (Figure 2b). We monitored the click reaction of BCN-N_3_ with alkynyl magnetic nanoparticles (MNPs) by UV–Vis spectrometer, and found that the characteristic absorbance of BCL-N_3_ in the supernatant at 263 nm dramatically decreased with an increasing reaction time until the reaction reached equilibrium after 2 h (Figure 3a), indicating the successful loading of BCL-N_3_ onto MNPs. Based on the amount of residual BCL-N_3_ in the supernatant of the reaction mixture, we calculated the BCL-N_3_ payload of MNPs was 36.7 nmol/mg.

The assembled BCL-N_3_@MNPs were further characterized by FT-IR and elementary analysis. In the FT-IR spectra of BCN-N_3_@MNPs (Figure 3b), the vibration absorption band at 562 cm^−1^ indicated the existence of Fe-O bonds, while the characteristic bands at 1435 cm^−1^ and 1083 cm^−1^ represented the existence of H-C-OH bonds and C-O-C bonds, respectively. The bands at 843 cm^−1^ and 1091 cm^−1^ showed the formation of an epoxy structure, which is the structural feature derived from the PEG linker in BCL-N_3_. In addition, the absorption bands at 2111 cm^−1^ indicated the formation of triazole ring, which resulted from the click reaction of the alkynyl group with azido group. The elementary analysis also supported the successful functionalization of MNPs by BCL-N_3_ (Figure 3c).

To further verify the assembling of baicalin functionalized nanoparticles, we used time-of-flight secondary mass spectrometry (ToF-SIMS) to characterize the BCL-N_3_@MNPs product which contains a triazole ring. As shown in Figure 3d, a negative ion peak at m/z 66.04 assignable to C_2_N_3_^−^, was observed in the spectrum of BCL-N_3_@MNP, whereas this ion peak was absent in the spectrum of BCL-N_3_, indicating the conjugation of BCL-N_3_ to alkynyl MNPs. We also characterized the morphology and size of BCL-N_3_@MNPs and MNPs by SEM. As shown in Figure 3e,f, the diameter of BCL-N_3_@MNPs was about 100 nm, almost no change in comparison with that of non-modified MNPs.

### 2.2. Evaluation of BCL-N3 Activity

**Figure 4:**
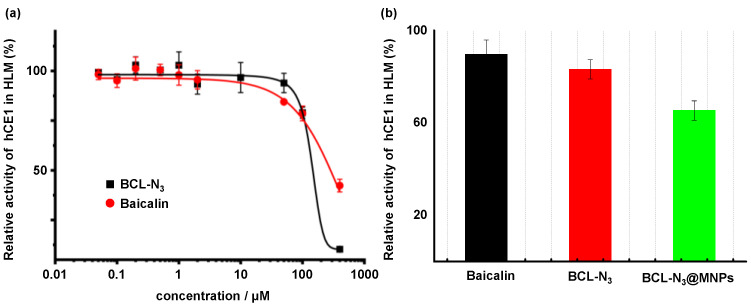
Evaluation of biological activity. (**a**) Inhibitory rate of baicalin and BCL-N_3_ towards hCE1 in HLMs; (**b**) relative inhibitory activity of 25 μM baicalin, BCL-N_3_ and BCL-N_3_@MNPs (counted on BCL-N_3_) towards hCE1 in HLMs.

In order to verify whether the azido modified baicalin retains the biological activity of baicalin, we compared the inhibitory activity of BCL-N_3_ on human carboxylesterase 1 (hCE1) in human liver microsomes (HLMs) with intact baicalin following a previously reported method [46]. We found that the azido modification via a PEG chain had little effect on the activity of baicalin. The IC_50_ values, which are the concentration of tested compounds achieving 50% inhibition on the hCE1 activity, of baicalin and BCL-N_3_ were calculated to be 98.1 ± 0.9 and 98.5 ± 1.4 μM, respectively. At the higher concentration (>100 μM), the inhibition potency of BCL-N_3_ was even higher than that of baicalin (Figure 4a). This may be ascribed to the introduction of a PEG side chain to baicalin which improved the water solubility of baicalin, thereby increasing its biological activity.

Unexpectedly, the baicalin functionalized MNPs, BCL-N_3_@MNPs, whose concentration was counted on BCL-N_3_, showed a higher inhibitory activity on hCE1 than baicalin and the azido baicalin (BCL-N_3_) at the same concentration (25 μM) (Figure 4b). It might be due to the influence of nanoparticles on the contact between the inhibitor, BCL-N_3_, and the hCE1 enzyme in HLMs. This implies the necessity of designing a control experiment to diminish the interference of nanoparticles on the capture of target proteins via non-specific absorption of proteins by use of intact MNPs (*vide infra*).

### 2.3. The Optimization of Capture of Target Proteins by BCL-N3@MNPs

**Figure 5:**
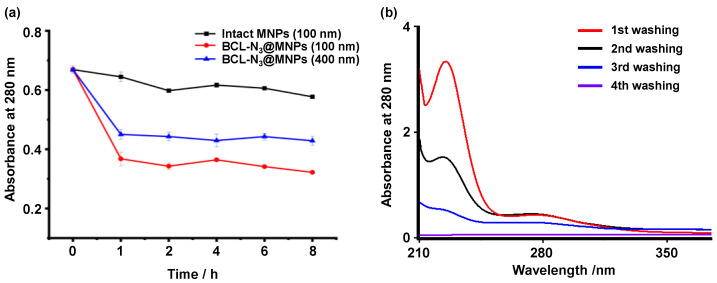
The optimization of protein capturing. (**a**) The absorbance at 280 nm of residual proteins in the supernatant of the mixtures of protein extracts from HEK293 cells incubated with different MNPs for various incubation times; (**b**) the absorbance at 280 nm of proteins washed off from 100 nm BCL-N_3_@MNPs which were incubated with protein extracts from HEK293 cells for 2 h.

To optimize the capture efficiency of BCL-N_3_@MNPs towards target proteins of baicalin, we compared the amount of proteins captured from protein extracts of human embryonic kidney (HEK293) cells by intact MNPs and BCL-N_3_@MNPs. As shown in Figure 5a, the amount of proteins in the supernatant, as indicated by the characteristic absorbance of proteins at 280 nm, dramatically decreased after the protein extract was incubated with 100 nm BCl-N_3_@MNPs for 1 h, whereas the amount of proteins in the supernatant only decreased slightly when the same amount of protein extract was incubated with 100 nm MNPs for 1 h, or even for 8 h. Moreover, we can see that 100 nm BCL-N_3_@MNPs captured more proteins than the 400 nm functionalized MNPs. This could be attributed to the larger surface area of the smaller nanoparticles, which were conferred higher efficiency to capture proteins. Hence, 100 nm BCL-N_3_@MNPs were used for subsequent experiments.

Although the intact MNPs showed much lower affinity to proteins in comparison with the functionalized MNPs, the non-specific absorption of proteins of nanoparticles was not negligible. In order to remove maximum nonspecific binding proteins from the nanoparticles, the BCL-N_3_@MNPs probes incubated with protein extracts for 2 h were washed four times by deionized water before the specifically bound proteins were eluted. As shown in Figure 5b, the large amount of proteins nonspecifically absorbed on BCL-N_3_@MNPs were removed after three times of washing, as indicated by the little absorption at 280 nm of the supernatant of the fourth washing. Under optimal conditions, i.e., 2 h incubation and three times of washing, the proteins captured by BCL-N_3_@MNPs (positive probe) and intact MNPs (negative probe) were determined to be 19.69 μg/mg and 4.71 μg/mg, respectively.

### 2.4. Target Proteins of Baicalin

**Figure 6:**
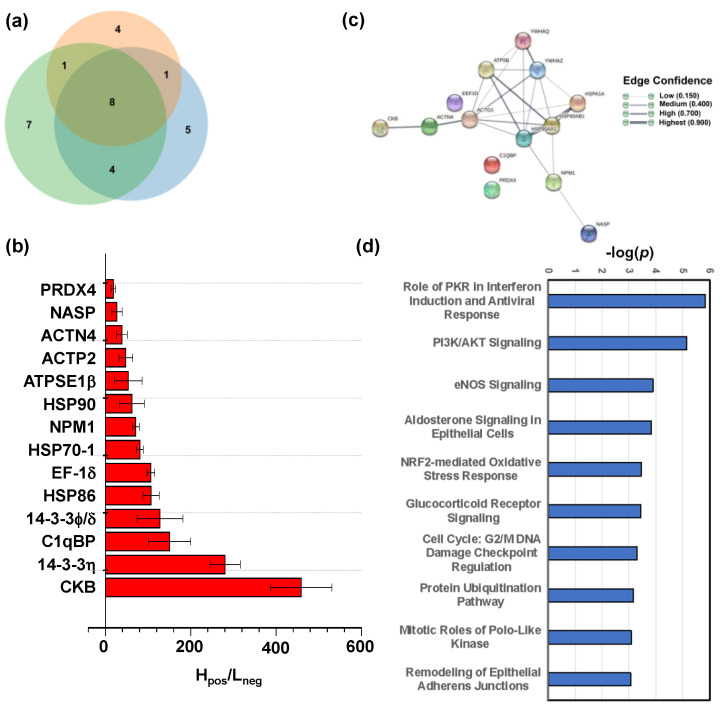
Identification of target proteins of baicalin. (**a**) Venn diagram of proteins captured by BCL-N_3_@MNPs (positive probe) and intact MNPs (negative probe) from protein extracts of HEK293 cells, and identified by mass spectrometric quantitation in three independent biological replicates. (**b**) The abundance ratios represented by H_pos_/L_neg_ determined by qMS analysis of the target proteins of baicalin; (**c**) protein–protein interaction (PPI) network of the target proteins generated by STRING 11.0; (**d**) top 10 core signaling pathways with which the target proteins of baicalin are highly associated. The higher the value of −log(*P*), the more closely associated the target proteins are with the signaling pathway.

Next, we used the baicalin functionalized nanoparticles (BCL-N_3_@MNPs) to capture proteins that interact with baicalin from the protein extract of HEK293 cells, and then identified the proteins by quantitative mass spectrometry (qMS) analysis following the method reported previously by us [32,39], while the intact MNPs were used as negative probes to rule out the proteins nonspecifically absorbed on MNPs. The protein fishing and nano LC-MS/MS analysis were performed in three replicates independently. In total, 14 proteins were identified in at least two parallel experiments (Figure 6a), and had an average ratio of H_pos_/L_neg_ > 18, where H_pos_ represents the sum of intensity of all tryptic peptides assigned to a protein captured by the positive probes, and L_neg_ indicates the sum of intensity of all tryptic peptides assigned to the same protein pull-down by the negative probes (Figure 6b). The detailed MS data of all identified proteins are listed in Table S1 in the Supplementary Materials. 

The proteins identified to interact with baicalin include five enzymes, three transcription regulators, one transporter, one kinase, one translation regulator and three other proteins, of which the gene ontology (GO) information are summarized in Table 1. Creatine kinase B type (CKB) protein had the strongest affinity with baicalin loaded on MNPs, as indicated by the highest H_pos_/L_neg_ ratio. This protein can reversibly catalyze the transfer of phosphate between ATP and various phosphagens [47]. Moreover, the ATP synthase subunit beta (ATPSF1β) located in mitochondrion, and three heat shock proteins (HSPs), HSP70-1, HSP90 and HSP86 which are all ATP-binding proteins and possess ATPase activity [48,49,50], were also identified to bind to baicalin (Figure 6b). Furthermore, one heat shock associated protein EF-1-δ [51] was also shown to have high affinity with baicalin. These results reveal that baicalin has high affinity to interact with ATP-binding and ATPase-activity proteins. 

As shown in the protein–protein interaction (PPI) network generated by STRING (Figure 6c), the three HSPs interact with each other as well as with the other five proteins, including adaptor proteins 14-3-3η and 14-3-3φ/δ which are encoded by *YWHAQ* [52] and *YWHAZ* [53], respectively, and also have a high affinity to baicalin. These interactions may contribute to the high H_pos_/L_neg_ ratio of the HSPs because most HSPs have molecular chaperone activity. With regard to this, we cannot exclude whether the HSPs interact with the adapter proteins or bind to baicalin directly.

Another target protein of baicalin with high H_pos_/L_neg_ ratio is C1qBP encoded by *C1QBP*. This protein is believed to be a multifunctional protein involved in inflammation and infection processes, ribosome biogenesis, protein synthesis in mitochondria, regulation of apoptosis, transcriptional regulation and pre-mRNA splicing [54,55,56,57,58,59,60]. The binding of baicalin to this protein may be related to its anti-inflammatory and anti-infection functions. 

The proteins NPM and NSAP are both nuclear proteins, and involved in diverse cellular processes such as protein chaperoning [61], ribosome biogenesis [62], cell proliferation, regulation of tumor suppressors p53/TP53 and ARF [63], DNA replication and normal cell cycle progression [64]. Given that the adapter proteins 14-3-3η and 14-3-3φ/δ are implicated in the positive regulation of protein insertion into the mitochondrial membrane involved in the apoptotic signaling pathway and in cellular component organization or biogenesis in cells, the interaction between baicalin and these four proteins may be related to the anticancer activity of baicalin. In addition, two actin related proteins (ACTP2, ACTN4) were revealed to interact with baicalin. Actin is a highly conserved protein that is involved in various types of cell motility [65,66]. The binding of baicalin to these actin-binding proteins may play a role in its various pharmacological activities, e.g., inhibiting cancer cell motility. Notably, the peroxiredoxin Prx-IV was found to interact with baicalin as well, though the H/L ratio was not high (Figure 6b). Prx-IV is a thiol-specific peroxidase, and catalyzes the reduction of hydrogen peroxide and organic hydroperoxides to water and alcohols [65,67], respectively. It plays a crucial role in cell protection against oxidative stress by detoxifying peroxides and as a sensor of hydrogen peroxide-mediated signaling events. The interaction of baicalin with this protein implicates it in the cellular redox homeostasis. 

**Figure 7:**
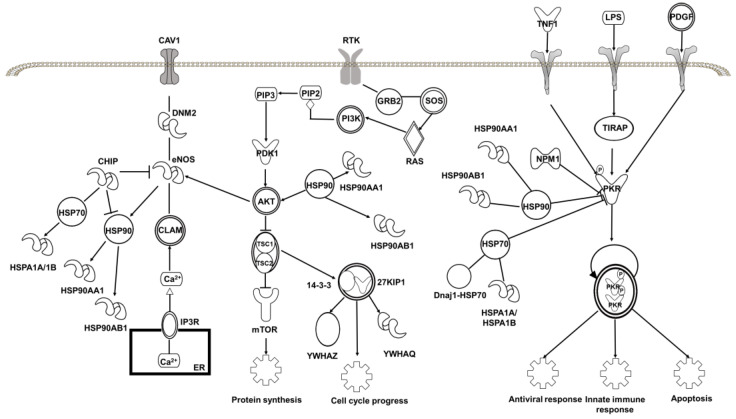
The top 3 core signaling pathways with which the target proteins of baicalin identified in this work are associated.

To further understand the biological activity of baicalin, we applied the Ingenuity Pathway Analysis (IPA) program to enrich the core signaling pathways with which the target proteins of baicalin are associated. The results showed that the target proteins are mostly associated with the role of PKR in interferon induction and antiviral response signaling pathway (*P* = 10^−6^) among the top 53 core signaling pathways (Figure 6d and Table S2). PKR is a double-stranded RNA-activated protein kinase, and can be activated by viral infection, playing a key role in the inflammasome [68] and in controlling viral spreading with the host [69]. The heat shock proteins, HSP90 and HSP70, and the nuclear protein NPM1 are all involved in regulation of PKR signaling (Figure 7). This implies that the binding of baicalin to these proteins may contribute to its antiviral and anti-inflammatory activities. 

Moreover, the target proteins of baicalin are highly associated with PI3K/AKT signaling pathway (*P* = 10^−5^) and eNOS signaling pathway (*P* = 10^−4^) (Figure 7). The abnormality of the PI3K/AKT pathway is common in cancer cells, and plays a crucial role in tumor transformation [70,71]. The endothelial nitric oxide synthase (eNOS-) signaling pathway, which produces NO and maintains an antiproliferative and antiapoptotic environment in cells [72], is regulated by diverse mechanisms including activation of the PI3K)/AKT pathway [73]. The involvement of baicalin-binding proteins, including the HSPs and the adapter proteins 14-3-3η and 14-3-3φ/δ, in these two signaling pathways likely contributes to its anticancer activity. This is consistent with the idea that the most associated disease with which the target proteins of baicalin are associated is cancer (Table S3). It is worth pointing out that the target proteins of baicalin are also closely related to organismal injury and abnormalities, and renal and urological disease, being in line with the clinical use of baicalin.

**Figure 8:**
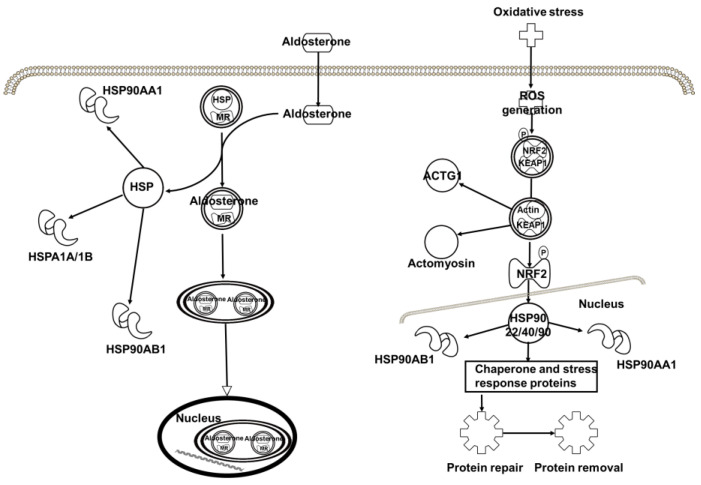
The top 4–5 core signaling pathways with which the target proteins of baicalin identified in this work are associated.

Interestingly, the baicalin-binding proteins, in particular HSPs, are also associated with the aldosterone signaling in epithelial cells and the NRF2-mediated oxidative stress response (Figure 8), indicating that baicalin processes an antioxidant function, which is supported by the interaction of baicalin with Prx-IV (*vide supra*).

## 3. Materials and Methods

### 3.1. Reagents and Materials

N-acetoxy-D_3_-succibinide (D_3_-NAS) and N-acetoxy-H_3_-succibinide (H_3_-NAS) were purchased from Sigma-Aldrich (St. Louis, MO, USA) and J & K Scientific (Shanghai, China), respectively. Trypsin and luciferin detection reagent were purchased from Promega (Beijing) Biotech Co., Ltd. (Beijing, China). Dithiothreitol (DTT) was obtained from Thermo Fisher (Thermo Fisher Scientific (China) Co., Ltd., Shanghai, China). Baicalin, alkyne-modified Fe_3_O_4_@SiO_2_ core-shell magnetic nanoparticles (30 mg/mL in 50% ethanol) and pooled human liver microsomes (HLMs) were purchased from Enriching Biotechnology Co., Ltd. (Shanghai, China), HWRK Chemical Co., Ltd. (Beijing, China) and RILD (Shanghai, China), respectively. The bioluminescent sensor D-Luciferin methyl ester (DME) [46] was a gift from Dr. Guangbo Ge at Dalian Institute of Chemical Physics, Chinese Academy of Sciences. Copper sulfate was purchased from Xilong Scientific Co., Ltd. (Shantou, China), sodium ascorbate, hydroxy benzotriazole (HOBT), 1-(3-dimethylaminopropyl) -3-ethylcarbodiimine hydrochloride (EDCI), and 2, 5-dihydroxybenzoic acid from Beijing Ouhe Technology Co., Ltd. (Beijing, China). ZiptipC18 was obtained from Millipore (Darmstadt, Germany). HEK293 cell line was purchased from National Experimental Cell Resource Sharing Platform (Beijing, China). BCA Protein Quantitative Kit was provided by Beyotime Biotechnology Co., Ltd. (Shanghai, China). DMEM containing 10% fetal bovine serum and 1% penicillin-streptomycin were purchased from Gibco (Grand Island, NY, USA). HPLC grade acetonitrile, colorless DMEM medium, trypsin-EDTA and fetal bovine serum were purchased from Invitrogen (Waltham, MA, USA). Deionized water produced by Millipore system was used throughout the experiments.

### 3.2. Instruments

Agilent 1200 high performance liquid chromatography (Agilent Technologies, Inc., Beijing, China), Bruker Avance III 400 HD NMR spectrometer (Bruker, Beijing, China) and Bruker Autoflex MALDI-TOF mass spectrometer (Bruker, Beijing, China) were used to purify and identify the target product, respectively. Bruker TENSOR-27 Infrared Spectroscope (Bruker, Beijing, China), UV-2550 UV–Vis spectrophotometer (Shimadzu Company, Kyoto, Japan) and IONTOF time of flight secondary ion mass spectrometer (ToF-SIMS V) (IONTOF, Munster, Germany) were used to characterize functionalized magnetic nanoprobes. Quantitative mass spectrometry analysis of proteins was performed on a Waters Xevo-G2-Q-TOF spectrometer (Waters, Milford, MA, USA), which was coupled to a Dionex nano-LC3500 System (Thermo Fisher Scientific (China) Co., Ltd., Shanghai, China). SYNERGY HIM Microplate reader (Bio Tek, Winowski, VT, USA) was used to measure the biological activity of baicalin and modified baicalin. 

### 3.3. Synthesis of Azido Modified Baicalin Derivative BCL-N3

Baicalin (1 mmol) and HOBT (1.3 mmol) were added to a round bottom flask, followed by addition of 6 mL anhydrous DMF, and then stirred for 30 min at room temperature. Aliquot of 11-azido-3,6,9-trioxaundecan-1 amine (1 mmol) and EDCI (1.3 mmol) were individually dissolved in 2 mL anhydrous DMF, and then successively added dropwise to the flask. After reaction for 20 min, excess of triethylamine (TEA) (3 mmol) was added, and the mixture was stirred at room temperature for 72 h. Finally, deionized water was added to stop the reaction, the product was extracted by CHCl_3_ three times, and the extractions were merged and dried by anhydrous magnesium sulfate overnight. With purification by silica gel column, we obtained the azido modified baicalin (BCL-N_3_) powder in light yellow (210.3 mg, yield 32.5%). ^1^H NMR (400 MHz, DMSO-*d_6_*) δ (ppm): 12.59 (br s,1H), 8.71 (s, 1H), 8.10 (d, *J* = 8 Hz, 2H), 8.04 (s, 1H), 7.61 (d, *J* = 8 Hz, 2H), 7.03 (d, 1H), 5.54 (d, 1H), 5.24 (d, *J* = 8 Hz, 1H), 5.10 (d, *J* = 8 Hz, 1H), 3.97 (d, J = 8 Hz, 1H), 3.45 (s, 4H), 3.42 (m, 10H), 3.41 (s, 6H), 1.23(s, 1H); ^13^C NMR (150 MHz, DMSO-*d_6_*) δ (ppm): 183.13, 168.62, 164.09, 151.89, 1489.77, 147.29, 132.65, 131.41, 1312, 129.71, 126.98, 106.77, 106.31, 101.11, 94.54, 76.16, 76.03, 73.32, 71.54, 70.27, 70.14, 69.77, 69.32, 50.52, 29.58. ESI-MS (m/z): C_29_H_34_N_4_O_13_ ([M + H]^+^), calculated: 647.21, found: 647.25. The ESI-MS and NMR spectra of BCL-N_3_ are shown in Figures S1–S3 in the Supplementary Materials. 

### 3.4. Preparation of Baicalin Functionalized Magnetic Nanoparticles BCL-N3@MNP

The newly synthesized azido modified baicalin (BCL-N_3_) described above was dissolved in DMSO to give rise to 5 mM stock solution which was diluted to requested concentration with PBS prior to use. The alkynyl functionalized magnetic nanoparticles (MNPs) were separated from 50% ethanol solution by an external magnetic field and washed three times with deionized water. Aliquot (100 μL, 3 mg) of the washed MNPs was dispersed into aqueous solution containing 1.5 mL BCL-N_3_ (1.5 μmol) in an Eppendorf tube by 1 min of sonication. Then, 2 μL of 50 mM sodium vitamin C and 2 μL of 5 mM anhydrous copper sulfate were added successively and incubated at room temperature for 1–8 h upon request. The baicalin functionalized probe (BCL-N_3_@MNP) was separated by magnetic separation, and characterized by UV–Vis, IR, elementary analysis and ToF-SIMS.

### 3.5. Measurement of Biological Activity of Azido-Modified Baicalin

The inhibitory activity of baicalin and azido-modified baicalin (BCL-N_3_) on human carboxylesterase 1 (hCE1) in human liver microsomes (HLMs) were measured by following a bioluminescence method reported previously [46] with modification. In brief, 20 μL of baicalin or BCL-N_3_ dissolved in PBS (100 mM, pH = 6.5) containing 1.25% DMSO at a series of concentrations, 0.125, 0.250, 0.500, 1.25, 2.5, 5, 12.5, 25, 125, 250 and 1000 μM, was pre-incubated with 20 μL of pooled HLMs at 37 °C for 30 min in a 96-well plate, followed by addition of 10 μL DME, the substrate of hCE1 at a final concentration of 3 μM for each well. Then, the plate was incubated at 37 °C for another 30 min for enzymatic hydrolysis of DME by hCE1, which was stopped by adding 50 μL of luciferin detection reagent (LDR), and the mixture was incubated at 37 °C for further 20 min. The bioluminescent intensity of each well was then measured by a microplate reader to calculate the inhibitory rate of tested compounds to hCE1 in comparison with that of control samples.

### 3.6. Cell Culturing and Protein Extraction

The embryonic kidney HEK293 cells were cultured in 90% DMEM (high glucose) supplemented with 10% fetal bovine serum and 1% penicillin-streptomycin in 100 mm dish under 5% CO_2_ at 37 °C. When cells covered 90% of the dish, the HEK293 cells were washed with cold PBS twice, centrifuged at 4 °C and 2500× *g* for 5 min. Then 5 × 10^6^–1 × 10^7^ cells were suspended in 500 μL of cold total protein extract solution from the Best-Bio Total Protein Extraction Kit (Bestbio Science, Nanjing, China) containing 2 μL protease inhibitors and 2 μL phosphatase inhibitors and shaken for 20 min on ice, and then centrifuged at 4 °C for 15 min. The supernatant containing soluble proteins were collected and stored at −80 °C prior to further use. The concentration of total proteins in the extracts was determined by BCA kit.

### 3.7. Quantitative Mass Spectrometry Analysis

The BCL-N_3_ functionalized MNPs (BCL-N_3_@MNPs, 10 mg) and equal amount of non-modified MNPs were individually incubated with 200 μg proteins extracted from HEK293 cells in PBS at 4 °C overnight. The nanoparticles were then separated by external magnetic force, and washed with PBS and deionized water three times each to remove nonspecific-binding proteins. The proteins captured by functionalized BCL-N_3_@MNPs probes and by MNPs probes were designated as positive samples and negative samples, respectively, and boiled at 95 °C for 5 min, followed by adding 50 μL of 100 mM DTT and incubating at 37 °C for 4 h, Thereafter, 100 μL of 40 mM IAA was added, and the samples were incubated at 37 °C for further 2 h, and then desalted by using 3K ultrafiltration tube at 14,000 g for 30 min. Finally, the proteins were digested with trypsin (*w*/*w*: 1:40) in 20 mM NH_4_HCO_3_ (pH 8.5) at 37 °C overnight, and the supernatant containing tryptic peptides was isolated by centrifuge for further use.

The peptides of each positive sample and negative sample were reacted with 100-fold excess of N-acetoxy-D_3_-succinimide (D_3_-NAS) and N-acetoxy-H_3_-succinimide (H_3_-NAS), respectively, at 37 °C under stirring for 5 h, and then mixed isovolumetrically, followed by addition of excess hydroxylamine and left to react for 20 min at pH 10–11 to remove excess D_3_-NAS and H_3_-NAS. Thereafter, the mixed heavy/light isotopic-labeled peptides were desalted by Ziptip C18 tips, and lyophilized prior to mass spectrometry analysis.

The desalted and lyophilized peptide mixture described above was re-dissolved in 5 μL deionized water containing 0.1% formic acid (FA) and injected into a Dionex Ultimated 3000 RSLCnano system. The peptides were concentrated by a C18 trap column (100 μm × 2 cm, 3 μm, Thermofisher Scientific), and then separated by a C18 column (50 μm × 10 cm, 2 μm, Thermofisher Scientific). The mobile phase A consisted of 0.1% formic acid, 4.9% acetonitrile and 95% deionized water, and mobile phase B consisted of 0.1% formic acid, 4.9% water and 95% acetonitrile. An isocratic flow at 5 μL/min for 10 min with solvent A alone was used for the peptide concentration and then a linear gradient from 1% to 45% solvent B over 120 min at a flow rate of 300 nL/min was used for the peptide separation. The RSLCnano system was coupled to a Xevo G2 QTOF electrospray ionization mass spectrometer (ESI-MS, Waters) equipped with a nanospray source. The MS data acquisition was performed in the data-dependent mode with a survey scan (*m*/*z* 350 to 1600) followed by MS/MS scans (*m*/*z* 50 to 2000) of the 8 most abundant precursor ions with charge states of 2+, 3+ or 4+. Precursor ions were filtered using a 20 s dynamic exclusion window. 

Proteins were identified by Mascot distiller 2.6.0 search engine based on the Swiss-Prot 201,604 (human) protein database. All of the spectra containing both mass peaks of D_3_- and H_3_-labeled peptides were combined to produce a composite MS spectrum. The mass tolerance was set as 50 ppm for precursor ion and 0.8 Da for product ions. Acetylation (Lys, N terminus) was set as fixed modification, and oxidation of methionine was chosen as variable modification. One missed cleavage by trypsin was allowed. Only “rank 1” (best match for each MS/MS mass spectrum) peptides were included. The intensity ratio of a D_3_-/H_3_-labeled peptide pair in the composite MS spectra was calculated from the sum of the peak heights of the three highest isotopic peaks. The relative abundance, represented by H_pos_/L_neg_, of a protein was calculated by averaging the intensity ratios of all peptides that matched to the protein. Statistics analyses were performed simultaneously by the Mascot distiller software (Matrix Science, Boston, MA, USA).

### 3.8. Bioinformatics Analysis

STRING (https://string-db.org/, 12 June 2022) was used to analyze the protein–protein interaction of the target proteins of baicalin identified by qMS analysis. Ingenuity Pathway Analysis (IPA, QIAGEN) [74] was used to enrich core pathways associated with the target proteins of baicalin. IPA maps each protein to the corresponding molecule in the Ingenuity Pathway Knowledge Base, which is available at the Ingenuity System’s web site (http://www.ingenuity.com, 6 June 2022).

## 4. Conclusions

In the present study, we designed and synthesized a clickable azido derivative of baicalin, BCL-N_3_, to functionalize alkynyl magnetic nanoparticles for assembling baicalin affinity nanoparticles (BCL-N_3_@MNPs), which were fully characterized by UV–Vis, IR, SEM and ToF-SIMS. The PEG chain between the azido group and the baicalin moiety enabled the azido derivative and the functionalized nanoparticles to preserve well the biological activity of baicalin. We then utilized an MS-based chemical proteomics strategy to identify the proteins enriched by BCL-N_3_@MNPs from the embryonic kidney HEK293 cells, and discovered 14 proteins interacting with baicalin. Among them were 6 ATP-binding proteins, including CKB, HSP86, HSP70-1, HSP90, ATPSF1β and ACTP2. Other baicalin binding proteins we identified include two adapter proteins 14-3-3η and 14-3-3φ/δ, an adaptive immunity responsible protein C1qBP, peroxiredoxin IV, the translation regulator EF-1-δ, the transcription regulator ACTN4, and nuclear proteins NPM and NASP. Bioinformatics analysis revealed that these baicalin-targeted proteins are involved mainly in the regulation of the role of PKR in interferon induction and the antiviral response signaling pathway, PI3K/AKT signaling pathway and eNOS signaling pathway, consistent with the pharmacological functions such as anti-inflammatory, antiviral and anticancer. These findings provide not only molecular evidence for the diverse biological activities of baicalin, but also a fundamental insight into further studies on the mechanism of action of baicalin. 

## 5. Patents

A Chinese patent, which documented the design and application of baicalin functionalized nanoparticles, has been granted in number ZL2017 1 0217509.5.

## Figures and Tables

**Table 1:** Proteins identified to interact with baicalin and the GO information of the proteins.

Gene Name	Protein Name (Abbreviation)	Protein Type	GO Information	
			Biological Process	Molecular Function
*CKB*	Creatine kinase B-type (CKB)	kinase	Creatine metabolism process, phosphocreatine biosynthesis	ATP binding, creatine kinase activity
*YWHAQ*	14-3-3 protein theta (14-3-3η)	enzyme	Negative regulation of ion transmembrane transport and transcription	14-3-3 protein binding, ion channel binding
*C1QBP*	Complement component 1 Q subcomponent-binding protein, mitochondrial (C1qBP)	transcription regulator	Adaptive immunity response, host-virusC1qBP interaction, mRNA splicing	Adrenergic receptor binding, complement component C1q complex binding
*YWHAZ*	14-3-3 protein zeta/delta (14-3-3φ/δ)	other	Adaptive immune response, cytokine-mediated signaling, Golgi reassembly	Cadherin binding, ion channel binding, protein kinase binding
*HSP90AA1*	Heat shock protein HSP 90-alpha (HSP86)	enzyme	Axon extension, response to heat, response to virus, chaperone mediated autophagy	ATPase activity, ATP binding, GTPase binding
*EEF1D*	Elongation factor 1-delta (EF-1-δ)	translation regulator	Cellular response to ionizing radiation, mRNA transcription	Activating transcription factor binding, cadherin binding, DNA binding
*HSPA1A*	Heat shock 70 kDa protein 1A, (HSP70-1)	enzyme	ATP metabolism, cellular heat acclimation, response to oxidative stress	ATPase activity, ATP binding, cadherin binding
*HSP90AB1*	Heat shock protein HSP 90-beta (HSP 90)	enzyme	Axon extension, response to heat, response to interleukin-4	ATPase activity, ATP binding, ATP-dependent protein binding
*NPM1*	Nucleophosmin (NPM)	transcription regulator	Cell aging, centrosome cycle, DNA repair, intracellular protein transport	Activating transcription factor binding, chromatin binding, RNA binding
*ATP5F1B*	ATP synthase subunit beta, (ATPSF1β)	transporter	ATP biosynthesis, lipid metabolism, mitochondrion organization	ATP binding, ATPase activity
*ACTG1*	Actin, cytoplasmic 2 (ACTP2)	other	Angiogenesis, positive regulation of cell migration, retina homeostasis	ATP binding, profiling binding
*ACTN4*	Alpha-actinin-4 (ACTN4)	transcription regulator	Protein transport, positive regulation of cell migration, platelet degranulation	Actin binding, calcium ion binding, chromatin DNA binding
*NASP*	Nuclear autoantigenic sperm protein (NASP)	other	Nucleosome assembly, DNA replication, histone exchange	Histone binding
*PRDX4*	Peroxiredoxin-4 (Prx-IV)	enzyme	Cell redox homeostasis, extracellular matrix organization, I-kappaB phosphorylation	Thioredoxin peroxidase activity

## Data Availability

Not applicable.

## Supplementary Materials

The following supporting information can be downloaded. Figure S1: ESI-MS spectrum of azido baicalin (BCL-N_3_); Figure S2: ^1^H NMR spectrum of azido baicalin (BCL-N_3_) in DMSO-*d_6_*; Figure S3: ^13^C NMR spectrum of azido baicalin (BCL-N_3_) in DMSO-*d_6_*; Table S1: MS quantitation data of target proteins of baicalin captured by BCL-N_3_@MNPs; Table S2: Core signaling pathways with which the target proteins of baicalin are associated; Table S3: Diseases and functions with which the target proteins of baicalin are associated.

## Sample Availability

Not available.
