# Supplementary material for: Baicalin Targets HSP70/90 to Regulate PKR/PI3K/AKT/eNOS Signaling Pathways
Source: Molecules. 2022 Feb 21;27(4):1432. doi: 10.3390/molecules27041432 (PMC8874410; doi:10.3390/molecules27041432)
Supplement: Supplementary file 1 [file molecules-27-01432-s001.zip › molecules-1597712-supplementary.pdf]

Baicalin targets HSP70/90 to regulate PKR/PI3K/AKT/eNOS signaling pathways

Yinzhu Hou[1,2,#], Zuqing Liang[1,2,#], Luyu Qi[1,2], Chao Tang[1], Xingkai Liu[1,2], Jilin Tang[1,2], Yao Zhao[1], Yanyan Zhang[1], Tiantian Fang[1], Qun Luo[1,2]*, Shijun Wang[3,]*, Fuyi Wang[1,2,3]*

1. Beijing National Laboratory for Molecular Sciences; CAS Research/Education Center for Excellence in Molecular Sciences; CAS Key Laboratory of Analytical Chemistry for Living Biosystems; National Centre for Mass Spectrometry in Beijing; Institute of Chemistry, Chinese Academy of Sciences, Beijing, 100190, People's Republic of China;
2. College of Chemical Science, University of Chinese Academy of Sciences, Beijing 100049, People's Republic of China;
3. College of Traditional Chinese Medicine, Shandong University of Traditional Chinese Medicine, Jinan 250355, People's Republic of China;

* Correspondence: fuyi.wang@iccas.ac.cn (FYW); wsj@sdutcm.edu.cn (SJW); qunluo@iccas.ac.cn (QL)

Supplementary Materials

Figure S1 – S3
Table S1 – S3

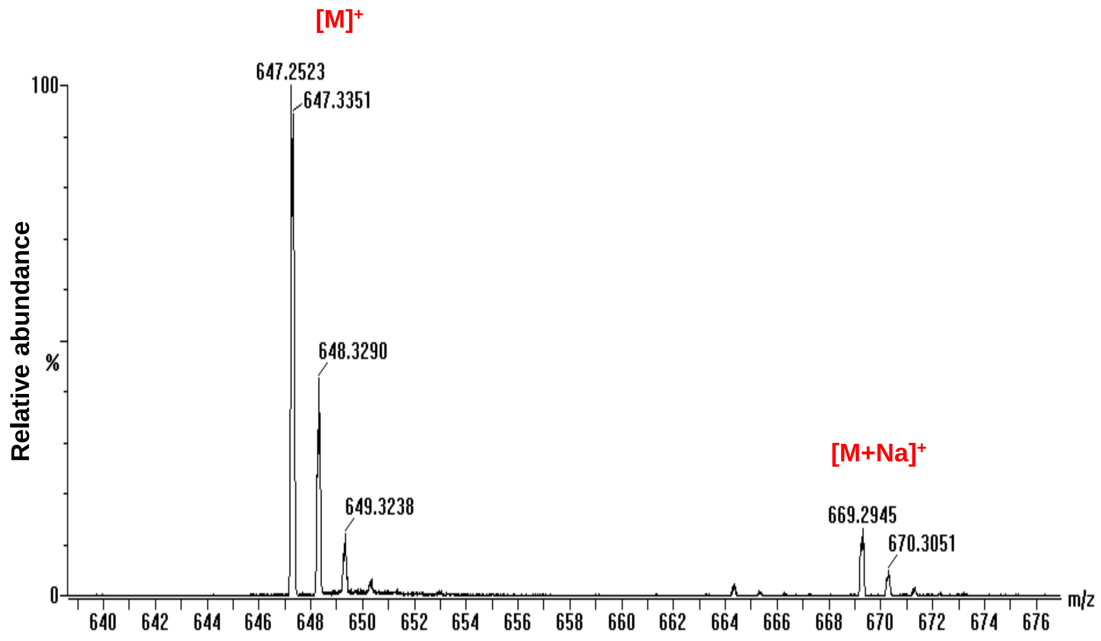

Figure S1. ESI-MS spectrum of azido baicalin (BCL-N_3).

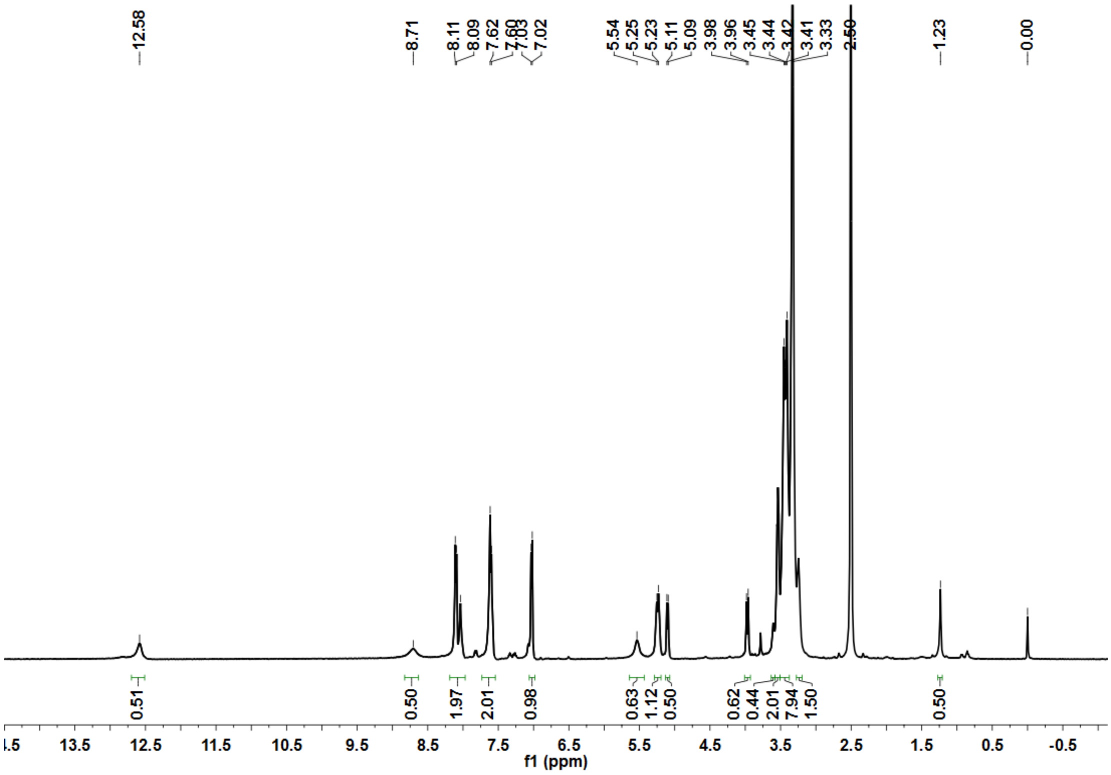

Figure S2. ^{1}H NMR spectrum of azido baicalin (BCL-N_3) in DMSO-d^6.

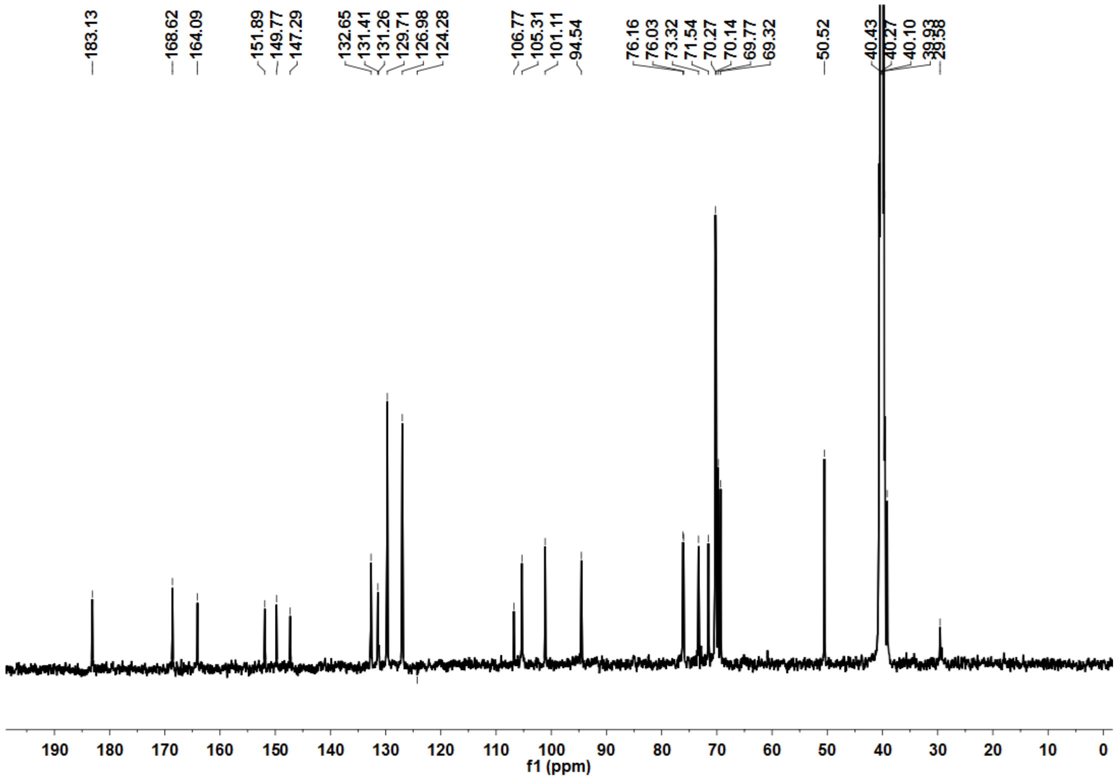

Figure S3. ^{13}C NMR spectrum of azido baicalin (BCL-N_3) in DMSO-d^6.

Table S1. MS quantitation data of target proteins of baicalin captured by BCL-N_3@MNPs.

prot_hit_num	prot_acc	prot_desc	H_{Pos}/ L_{Neg}	prot_cover (%)	pep_exp_mz	pep_exp_mr	pep_exp_z	pep_calc_mr	pep_delta	pep_miss	pep_expect	pep_seq	pep_var_mod
**1**	CKB	Creatine kinase B-type OS=Homo sapiens OX=9606 GN=CKB PE=1 SV=1	458.5±35	(1)31	811.4134	1620.8122	2	1619.836	0.9762	1	3.2	MPFSNSHNALKLR	Oxidation (M); Acetyl:2H(3) (N-term); Acetyl:2H(3) (K)
					816.4394	1630.8643	2	1630.8605	0.0038	0	6.50E-05	LAVEALSSLDGDLAGR	Acetyl:2H(3) (N-term)
					970.0232	1938.0318	2	1938.0291	0.0027	0	9.00E-05	LGFSEVELVQMVVDGVK	Acetyl:2H(3) (N-term); Acetyl:2H(3) (K)
					978.0209	1954.0273	2	1954.024	0.0033	0	0.00032	LGFSEVELVQMVVDGVK	Oxidation (M); Acetyl:2H(3) (N-term); Acetyl:2H(3) (K)
					1005.4887	2008.9628	2	2008.953	0.0099	0	1.10E-09	GTGGVDTAAVGGVFDVSNADR	Acetyl:2H(3) (N-term)
					849.411	2545.2112	3	2544.1974	1.0138	1	3.9	LRFPAEDEFPDLSAHNNHMAK	Oxidation (M); Acetyl:2H(3) (N-term); Acetyl:2H(3) (K)
					1237.5841	3709.7305	3	3708.8286	0.9019	0	0.26	SMTEAEQQQLIDDHFLFDKPVSPLLLASGMAR	2 Oxidation (M); Acetyl:2H(3) (N-term); Acetyl:2H(3) (K)
				(2)23.6	816.4469	1630.8793	2	1630.8605	0.0188	0	8.70E-08	LAVEALSSLDGDLAGR	Acetyl:2H(3) (N-term)

					851.9448	1701.875	2	1701.8554	0.0196	0	0.0003	TFLVWVNEEDHLR	Acetyl:2H(3) (N-term)
					970.0322	1938.0498	2	1938.0291	0.0207	0	9.50E-06	LGFSEVELVQMVVDGVK	Acetyl:2H(3) (N-term); Acetyl:2H(3) (K)
					978.03	1954.0455	2	1954.024	0.0215	0	0.00012	LGFSEVELVQMVVDGVK	Oxidation (M); Acetyl:2H(3) (N-term); Acetyl:2H(3) (K)
					1005.9947	2009.9748	2	2008.953	1.0219	0	2.10E-08	GTGGVDTAAVGGVFDVSNADR	Acetyl:2H(3) (N-term)
					854.3982	2560.1729	3	2559.1725	1.0004	0	2.2	TDLNPDNLQGGDDLDPNYVLSSR	Acetyl (N-term)
				(3)19.7	816.4394	1630.8643	2	1630.8605	0.0037	0	1.90E-05	LAVEALSSLDGDLAGR	Acetyl:2H(3) (N-term)
					970.0253	1938.036	2	1938.0291	0.0069	0	6.90E-05	LGFSEVELVQMVVDGVK	Acetyl:2H(3) (N-term); Acetyl:2H(3) (K)
					978.0178	1954.0211	2	1954.024	-0.0029	0	0.0019	LGFSEVELVQMVVDGVK	Oxidation (M); Acetyl:2H(3) (N-term); Acetyl:2H(3) (K)
					1005.4885	2008.9624	2	2008.953	0.0095	0	4.50E-10	GTGGVDTAAVGGVFDVSNADR	Acetyl:2H(3) (N-term)
					847.0812	2538.2219	3	2538.1597	0.0621	1	2.3	LRFPAEDEFPDLSAHNNHMAK	Oxidation (M); Acetyl (N-term); Acetyl (K)
**2**	YWHAQ	14-3-3 protein theta OS=Homo	280.4±72	(1)16.3	802.4851	801.4778	1	801.4649	0.013	1	0.63	NVVGGRR	Acetyl:2H(3) (N-term)

sapiens OX=9606 GN=YWHAQ PE=1 SV=1										
		585.2965	1168.5785	2	1168.5738	0.0047	0	0.012	EMQPTHPIR	Oxidation (M); Acetyl:2H(3) (N-term)
		617.8495	1233.6844	2	1233.683	0.0014	0	7.80E-06	DSTLIMQLLR	Acetyl:2H(3) (N-term)
		625.842	1249.6695	2	1249.6779	-0.0084	0	1.1	DSTLIMQLLR	Oxidation (M); Acetyl:2H(3) (N-term)
		789.3792	1576.7439	2	1576.7408	0.0031	0	7.90E-07	AVTEQGAELSNEER	Acetyl:2H(3) (N-term)
	(2)20	496.2696	990.5246	2	990.5386	-0.0139	0	2.7	NLLSVAYK	Acetyl (N-term); Acetyl (K)
		497.2952	992.5758	2	992.5661	0.0098	0	0.0024	VISSIEQK	Acetyl:2H(3) (N-term); Acetyl:2H(3) (K)
		585.7867	1169.5588	2	1168.5738	0.985	0	0.089	EMQPTHPIR	Oxidation (M); Acetyl:2H(3) (N-term)
		617.8544	1233.6943	2	1233.683	0.0113	0	7.30E-05	DSTLIMQLLR	Acetyl:2H(3) (N-term)
		625.8528	1249.6909	2	1249.6779	0.013	0	6.50E-05	DSTLIMQLLR	Oxidation (M); Acetyl:2H(3) (N-term)
		789.3868	1576.7589	2	1576.7408	0.0182	0	7.30E-07	AVTEQGAELSNEER	Acetyl:2H(3) (N-term)
	(3)16.7	585.7809	1169.5473	2	1168.5738	0.9735	0	0.33	EMQPTHPIR	Oxidation (M); Acetyl:2H(3) (N-term)
		617.8491	1233.6836	2	1233.683	0.0006	0	8.10E-06	DSTLIMQLLR	Acetyl:2H(3) (N-term)

					625.8468	1249.6791	2	1249.6779	0.0012	0	5.40E-06	DSTLIMQLLR	Oxidation (M); Acetyl:2H(3) (N-term)
					789.3806	1576.7467	2	1576.7408	0.0059	0	1.50E-08	AVTEQGAELSN EER	Acetyl:2H(3) (N-term)
					836.0863	2505.2371	3	2504.2394	0.9977	1	2.8	AVTEQGAELSN EERNLLSVAYK	Acetyl (N-term); Acetyl (K)
**3**	HSPA1 A	Heat shock 70 kDa protein 1A OS=Homo sapiens OX=9606 GN=HSPA1 A PE=1 SV=1	106.4±8.7	(1)29.8	629.8005	1257.5864	2	1257.6089	-0.0224	1	2.1	SAVEDEGLKGK	Acetyl (N-term); 2 Acetyl (K)
					634.7858	1267.5571	2	1266.5802	0.9769	1	0.48	MVQEAEKYK	Oxidation (M); Acetyl (N-term); 2 Acetyl (K)
					637.3337	1272.6529	2	1272.6501	0.0028	0	4.30E-06	VEIIANDQGNR	Acetyl:2H(3) (N-term)
					755.9271	1509.8396	2	1509.8343	0.0053	0	1.80E-05	AQIHDLVLVGG STR	Acetyl:2H(3) (N-term)
					766.8718	1531.7291	2	1531.7234	0.0057	0	0.00031	TTPSYVAFTDT ER	Acetyl:2H(3) (N-term)
					856.0073	1710	2	1708.9472	1.0528	1	2.5	QATKDAGVIAG LNVLR	Acetyl (N-term); Acetyl (K)
					861.3959	1720.7773	2	1719.7528	1.0245	0	0.36	ATAGDTHLGGE DFDNR	Acetyl:2H(3) (N-term)

		781.6851	2342.0335	3	2341.0784	0.9552	1	0.42	NALESYAFNMKSAVEDEGLK	Acetyl (N-term); 2 Acetyl (K)
		944.8062	2831.3966	3	2830.3853	1.0113	0	0.00024	QTQIFTTYSDNQPGVLIQVYEGER	Acetyl:2H(3) (N-term)
		1016.5151	3046.5235	3	3045.5163	1.0072	0	0.73	EIAEAYLGYPVTNAVITVPAYFNDSQR	Acetyl:2H(3) (N-term)
		1091.2748	3270.8026	3	3269.7951	1.0075	0	0.0081	SENVQDLLLLDVAPLSLGLETAGGVMTALIK	Acetyl:2H(3) (N-term); Acetyl:2H(3) (K)
		1096.6017	3286.7833	3	3285.79	0.9933	0	0.014	SENVQDLLLLDVAPLSLGLETAGGVMTALIK	Oxidation (M); Acetyl:2H(3) (N-term); Acetyl:2H(3) (K)
		1146.2887	3435.8443	3	3435.8535	-0.0092	1	0.61	SENVQDLLLLDVAPLSLGLETAGGVMTALIKR	Oxidation (M); Acetyl (N-term); Acetyl (K)
		1206.9266	3617.758	3	3616.8168	0.9412	1	0.061	NSTIPTKQTQIFTTYSDNQPGVLIQVYEGER	Acetyl:2H(3) (N-term); Acetyl:2H(3) (K)
	(2)24	622.3536	1242.6927	2	1241.7171	0.9756	0	0.038	DAGVIAGLNVLR	Acetyl:2H(3) (N-term)
		637.339	1272.6634	2	1272.6501	0.0133	0	0.00028	VEIIANDQGNR	Acetyl:2H(3) (N-term)
		755.933	1509.8514	2	1509.8343	0.0171	0	0.00078	AQIHDLVLVGGSTR	Acetyl:2H(3) (N-term)

	766.8774	1531.7403	2	1531.7234	0.0169	0	0.0019	TTPSYVAFTDTER	Acetyl:2H(3) (N-term)
	568.9649	1703.8729	3	1703.8599	0.013	0	2.6	AFYPEEISSMVLTK	Acetyl:2H(3) (N-term); Acetyl:2H(3) (K)
	853.4172	1704.8199	2	1703.8599	0.9601	0	2.6	AFYPEEISSMVLTK	Acetyl:2H(3) (N-term); Acetyl:2H(3) (K)
	1016.5246	3046.552	3	3045.5163	1.0357	0	1.80E-05	EIAEAYLGYPVTNAVITVPAYFNDSQR	Acetyl:2H(3) (N-term)
	628.8955	3139.4413	5	3138.5081	0.9332	0	2.1	ELEQVCNPIISGLYQGAGGPGPGGFGAQGPK	Acetyl (N-term); Acetyl (K)
	1090.95	3269.8282	3	3269.7951	0.0331	0	8.40E-05	SENVQDLLLLDVAPLSLGLETAGGVMTALIK	Acetyl:2H(3) (N-term); Acetyl:2H(3) (K)
	859.9753	3435.8721	4	3435.8535	0.0186	1	0.14	SENVQDLLLLDVAPLSLGLETAGGVMTALIKR	Oxidation (M); Acetyl (N-term); Acetyl (K)
(3)35.6	421.2555	840.4964	2	839.4699	1.0265	1	1.7	STGKANK	Acetyl:2H(3) (N-term); 2 Acetyl:2H(3) (K)
	577.8058	1153.5971	2	1153.5959	0.0012	0	0.21	LLQDFFNGR	Acetyl:2H(3) (N-term)
	620.3688	1238.7231	2	1238.6983	0.0248	0	20	DAGVIAGLNVLR	Acetyl (N-term)
	629.8014	1257.5882	2	1257.6089	-0.0207	1	1.3	SAVEDEGLKGK	Acetyl (N-term); 2 Acetyl (K)
	637.3339	1272.6533	2	1272.6501	0.0032	0	0.00026	VEIIANDQGNR	Acetyl:2H(3) (N-term)

	638.8306	1275.6466	2	1275.6367	0.0099	1	4.5	MVQEAEKYK	Oxidation (M); Acetyl:2H(3) (N-term); 2 Acetyl:2H(3) (K)
	662.3074	1322.6003	2	1321.586	1.0143	0	2.4	FGDPVVQSDM K	Oxidation (M); Acetyl (N-term); Acetyl (K)
	743.8915	1485.7684	2	1484.7531	1.0153	1	3	KFGDPVVQSD MK	Acetyl:2H(3) (N-term); 2 Acetyl:2H(3) (K)
	755.9268	1509.8391	2	1509.8343	0.0049	0	2.90E-05	AQIHDLVLVGG STR	Acetyl:2H(3) (N-term)
	766.8722	1531.7298	2	1531.7234	0.0064	0	0.0014	TTPSYVAFTDT ER	Acetyl:2H(3) (N-term)
	861.3857	1720.7569	2	1719.7528	1.0041	0	0.39	ATAGDTHLGGE DFDNR	Acetyl:2H(3) (N-term)
	921.7645	2762.2716	3	2762.3408	-0.0692	1	1.8	MAKAAAIGIDL GTTYSCVGVFQ HGK	Acetyl (Protein N-term); Acetyl (N-term); 2 Acetyl (K)
	944.4719	2830.394	3	2830.3853	0.0087	0	7.90E-07	QTQIFTTYSDN QPGVLIQVYEG ER	Acetyl:2H(3) (N-term)
	1016.1791	3045.5155	3	3045.5163	-0.0008	0	0.00026	EIAEAYLGYPV TNAVITVPAYF NDSQR	Acetyl:2H(3) (N-term)
	1090.9405	3269.7997	3	3269.7951	0.0046	0	0.021	SENVQDLLLLD VAPLSLGLETA GGVMTALIK	Acetyl:2H(3) (N-term); Acetyl:2H(3) (K)

					1206.5901	3616.7485	3	3616.8168	-0.0683	1	0.0094	NSTIPTKQTQIFTTYSDNQPGVLIQVYEGER	Acetyl:2H(3) (N-term); Acetyl:2H(3) (K)
**4**	C1QBP	Complement component 1 Q subcomponent-binding protein, mitochondrial OS=Homo sapiens OX=9606 GN=C1QBP PE=1 SV=1	150.42±49 (n=2)	(1)43.3	482.7887	963.5629	2	963.5501	0.0128	0	2.4	QLLQPAPR	Acetyl (N-term)
					864.9236	1727.8327	2	1726.7991	1.0337	0	0.98	MSGGWELELNGTEAK	Oxidation (M); Acetyl:2H(3) (N-term); Acetyl:2H(3) (K)
					894.4538	1786.893	2	1786.8896	0.0035	1	4.10E-07	AFVDFLSDEIKEER	Acetyl:2H(3) (N-term); Acetyl:2H(3) (K)
					1107.0314	2212.0482	2	2211.1079	0.9403	1	0.69	TLPKMSGGWELELNGTEAK	Oxidation (M); Acetyl:2H(3) (N-term); 2 Acetyl:2H(3) (K)
					1472.218	2942.4214	2	2941.4081	1.0134	1	0.86	VEEQEPELTSTPNFVVEVIKNDDGK	Acetyl (N-term); 2 Acetyl (K)

	1582.73	3163.4454	2	3162.432	1.0135	0	8.10E-09	DTNYTLNTDSL DWALYDHLMD FLADR	Acetyl:2H(3) (N-term)
	1060.8286	3179.464	3	3178.4269	1.0371	0	0.76	DTNYTLNTDSL DWALYDHLMD FLADR	Oxidation (M); Acetyl:2H(3) (N-term)
	1177.2522	3528.7348	3	3528.7306	0.0041	0	0.014	GVDNTFADELV ELSTALEHQEYI TFLEDLK	Acetyl:2H(3) (N-term); Acetyl:2H(3) (K)
(2)35.8	789.1296	2364.3669	3	2363.326	1.0409	1	2.2	QLLQPAPRLCT RPFGLLSVR	Acetyl (N-term)
	984.8254	2951.4545	3	2950.4645	0.99	1	1.5	VEEQEPELTSTP NFVVEVIKNDD GK	Acetyl:2H(3) (N-term); 2 Acetyl:2H(3) (K)
	1055.1622	3162.4648	3	3162.432	0.0328	0	4.20E-07	DTNYTLNTDSL DWALYDHLMD FLADR	Acetyl:2H(3) (N-term)
	1582.7424	3163.4702	2	3162.432	1.0383	0	1.50E-09	DTNYTLNTDSL DWALYDHLMD FLADR	Acetyl:2H(3) (N-term)
	1060.495	3178.4632	3	3178.4269	0.0363	0	6.10E-08	DTNYTLNTDSL DWALYDHLMD FLADR	Oxidation (M); Acetyl:2H(3) (N-term)
	1177.263	3528.7672	3	3528.7306	0.0365	0	0.0032	GVDNTFADELV ELSTALEHQEYI TFLEDLK	Acetyl:2H(3) (N-term); Acetyl:2H(3) (K)

**5**	HSP90 AB1	Heat shock protein HSP 90-beta OS=Homo sapiens OX=9606 GN=HSP90AB1 PE=1 SV=4	62.05±29	(1)21.4	412.7471	823.4796	2	822.4969	0.9826	0	1.4	SLVSVTK	Acetyl:2H(3) (N-term); Acetyl:2H(3) (K)
					496.2966	990.5786	2	990.5769	0.0016	0	0.021	TKPIWTR	Acetyl:2H(3) (N-term); Acetyl:2H(3) (K)
					582.7715	1163.5284	2	1163.5499	-0.0215	0	1.8	APFDLFENK	Acetyl (N-term); Acetyl (K)
					662.8026	1323.5905	2	1323.6203	-0.0297	1	2.3	FENLCKLMK	Oxidation (M); Acetyl (N-term); 2 Acetyl (K)
					666.8867	1331.7589	2	1331.7567	0.0022	0	1.3	ADLINNLGTIAK	Acetyl:2H(3) (N-term); Acetyl:2H(3) (K)
					675.252	1348.4894	2	1347.5145	0.9749	0	1	DNSTMGYMMAK	Oxidation (M); Acetyl (N-term); Acetyl (K)
					678.8052	1355.5958	2	1355.592	0.0038	0	0.0013	EDQTEYLEER	Acetyl:2H(3) (N-term)
					720.38	1438.7455	2	1438.786	-0.0405	0	2.6	TLTLVDTGIGMTK	Acetyl:2H(3) (N-term); Acetyl:2H(3) (K)
					764.8323	1527.65	2	1526.6765	0.9735	1	0.99	DNSTMGYMMAKK	Oxidation (M); Acetyl:2H(3) (N-term); 2 Acetyl:2H(3) (K)

		876.5145	1751.0144	2	1749.9823	1.0321	1	1.2	ADLINNLGTIAK SGTK	Acetyl:2H(3) (N-term); 2 Acetyl:2H(3) (K)
		835.3378	2502.9914	3	2503.0246	-0.0331	1	3.4	DDEEKPKIEDV GSDEEDDSGK	Acetyl (N-term); 3 Acetyl (K)
		908.8177	2723.4312	3	2723.4977	-0.0665	1	1.1	TLTLVDTGIGM TKADLINNLGTI AK	Oxidation (M); Acetyl:2H(3) (N-term); 2 Acetyl:2H(3) (K)
		969.1038	2904.2896	3	2903.3326	0.957	1	1.1	LVSSPCCIVTST YGWTANMERI MK	Oxidation (M); Acetyl (N-term); Acetyl (K)
		1011.8607	3032.5603	3	3032.5534	0.0069	0	3.50E-05	DLVVLLFETAL LSSGFSLEDPQT HSNR	Acetyl:2H(3) (N-term)
	(2)21	412.7507	823.4869	2	822.4969	0.99	0	1.2	SLVSVTK	Acetyl:2H(3) (N-term); Acetyl:2H(3) (K)
		496.3016	990.5887	2	990.5769	0.0117	0	0.054	TKPIWTR	Acetyl:2H(3) (N-term); Acetyl:2H(3) (K)
		499.2372	996.4599	2	995.4864	0.9735	0	0.43	ADHGEPIGR	Acetyl:2H(3) (N-term)
		620.3492	1238.6839	2	1238.6698	0.0141	0	0.83	IDIIPNPQER	Acetyl:2H(3) (N-term)
		666.8917	1331.7689	2	1331.7567	0.0122	0	0.1	ADLINNLGTIAK	Acetyl:2H(3) (N-term); Acetyl:2H(3) (K)
		675.2584	1348.5023	2	1347.5145	0.9878	0	0.83	DNSTMGYMMA K	Oxidation (M); Acetyl (N-term); Acetyl (K)
		720.409	1438.8034	2	1438.786	0.0174	0	0.00021	TLTLVDTGIGM TK	Acetyl:2H(3) (N-term); Acetyl:2H(3) (K)

	779.9193	1557.8239	2	1557.8078	0.0162	0	0.00024	GVVDSEDLPLN ISR	Acetyl:2H(3) (N-term)
	831.7064	2492.0974	3	2492.1214	-0.0239	0	1.5	LVSSPCCIVTST YGWTANMER	Oxidation (M); Acetyl:2H(3) (N-term)
	846.3835	2536.1286	3	2535.0894	1.0393	1	1.6	YHTSQSGDEMT SLSEYVSRMK	Oxidation (M); Acetyl (N-term); Acetyl (K)
	909.1472	2724.4198	3	2723.4977	0.9221	1	0.78	TLTLVDTGIGM TKADLINNLGTI AK	Oxidation (M); Acetyl:2H(3) (N-term); 2 Acetyl:2H(3) (K)
	1011.8686	3032.584	3	3032.5534	0.0306	0	2.80E-07	DLVVLLFETAL LSSGFSLEDPQT HSNR	Acetyl:2H(3) (N-term)
	1517.8027	3033.5908	2	3032.5534	1.0374	0	1.80E-08	DLVVLLFETAL LSSGFSLEDPQT HSNR	Acetyl:2H(3) (N-term)
(3)27.2	412.7474	823.4803	2	822.4969	0.9833	0	1.5	SLVSVTK	Acetyl:2H(3) (N-term); Acetyl:2H(3) (K)
	437.7837	873.5529	2	873.5515	0.0014	0	0.0019	ALLFIPR	Acetyl:2H(3) (N-term)
	496.2963	990.5781	2	990.5769	0.0012	0	0.01	TKPIWTR	Acetyl:2H(3) (N-term); Acetyl:2H(3) (K)
	498.7515	995.4885	2	995.4864	0.0021	0	0.57	ADHGEPIGR	Acetyl:2H(3) (N-term)
	620.3436	1238.6726	2	1238.6698	0.0028	0	0.014	IDIIPNPQER	Acetyl:2H(3) (N-term)
	655.3234	1308.6322	2	1307.6254	1.0068	1	1.7	FENLCKLMK	Acetyl (N-term); 2 Acetyl (K)
	662.8019	1323.5893	2	1323.6203	-0.031	1	11	FENLCKLMK	Oxidation (M); Acetyl (N-term); 2 Acetyl (K)

	664.3641	1326.7137	2	1325.7191	0.9947	0	3.8	ADLINNLGTIAK	Acetyl (N-term); Acetyl (K)
	666.8869	1331.7592	2	1331.7567	0.0025	0	0.09	ADLINNLGTIAK	Acetyl:2H(3) (N-term); Acetyl:2H(3) (K)
	675.2494	1348.4843	2	1347.5145	0.9698	0	0.93	DNSTMGYMMAK	Oxidation (M); Acetyl (N-term); Acetyl (K)
	678.8062	1355.5977	2	1355.592	0.0058	0	1.80E-06	EDQTEYLEER	Acetyl:2H(3) (N-term)
	720.4018	1438.789	2	1438.786	0.003	0	0.25	TLTLVDTGIGMTK	Acetyl:2H(3) (N-term); Acetyl:2H(3) (K)
	764.834	1527.6534	2	1526.6765	0.9769	1	4.7	DNSTMGYMMAKK	Oxidation (M); Acetyl:2H(3) (N-term); 2 Acetyl:2H(3) (K)
	779.9135	1557.8125	2	1557.8078	0.0047	0	3.40E-07	GVVDSEDLPLNISR	Acetyl:2H(3) (N-term)
	790.332	1578.6495	2	1577.6217	1.0278	0	1.9	IEDVGSDEEDDSGK	Acetyl (N-term); Acetyl (K)
	809.9085	1617.8023	2	1616.7953	1.007	0	0.0021	SLTNDWEDHLAVK	Acetyl:2H(3) (N-term); Acetyl:2H(3) (K)
	637.3009	1908.8809	3	1908.865	0.0158	1	5.9	AQALRDNSTMGYMMAK	2 Oxidation (M); Acetyl:2H(3) (N-term); Acetyl:2H(3) (K)
	820.4234	2458.2484	3	2457.2057	1.0427	1	4	GVVDSEDLPLNISREMLQQSK	Oxidation (M); Acetyl (N-term); Acetyl (K)
	846.3769	2536.109	3	2535.0894	1.0197	1	1.7	YHTSQSGDEMTSLSEYVSRMK	Oxidation (M); Acetyl (N-term); Acetyl (K)

					1011.8613	3032.5621	3	3032.5534	0.0087	0	1.60E-05	DLVVLLFETAL LSSGFSLEDPQT HSNR	Acetyl:2H(3) (N-term)
**6**	YWH AZ	14-3-3 protein zeta/delta OS=Homo sapiens OX=9606 GN=YWHAZ PE=1 SV=1	127.9±54	(1)38.8	585.2965	1168.5785	2	1168.5738	0.0047	0	0.012	EMQPTHPIR	Oxidation (M); Acetyl:2H(3) (N-term)
					617.8495	1233.6844	2	1233.683	0.0014	0	7.80E-06	DSTLIMQLLR	Acetyl:2H(3) (N-term)
					618.7996	1235.5846	2	1234.5717	1.0129	0	6.5	YLAEVAAGDD K	Acetyl (N-term); Acetyl (K)
					625.842	1249.6695	2	1249.6779	-0.0084	0	1.1	DSTLIMQLLR	Oxidation (M); Acetyl:2H(3) (N-term)
					499.2764	1494.8074	3	1494.7949	0.0125	1	0.42	SSWRVVSSIEQ K	Acetyl:2H(3) (N-term); Acetyl:2H(3) (K)
					797.3808	1592.747	2	1592.7357	0.0113	0	1.30E-07	SVTEQGAELSN EER	Acetyl:2H(3) (N-term)
					673.2682	2016.7829	3	2016.8227	-0.0398	1	3.5	LAEQAERYDD MAACMK	2 Oxidation (M); Acetyl (N-term); Acetyl (K)
					819.0223	2454.045	3	2453.0182	1.0268	0	1.6	DNLTLWTSDTQ GDEAEAGEGGE N	Acetyl:2H(3) (N-term)

	(2)38.4	496.2696	990.5246	2	990.5386	-0.0139	0	2.7	NLLSVAYK	Acetyl (N-term); Acetyl (K)
		585.7867	1169.5588	2	1168.5738	0.985	0	0.089	EMQPTHPIR	Oxidation (M); Acetyl:2H(3) (N-term)
		617.8544	1233.6943	2	1233.683	0.0113	0	7.30E-05	DSTLIMQLLR	Acetyl:2H(3) (N-term)
		618.8036	1235.5926	2	1234.5717	1.0209	0	0.64	YLAEVAAGDDK	Acetyl (N-term); Acetyl (K)
		625.8528	1249.6909	2	1249.6779	0.013	0	6.50E-05	DSTLIMQLLR	Oxidation (M); Acetyl:2H(3) (N-term)
		797.3878	1592.761	2	1592.7357	0.0253	0	1.40E-06	SVTEQGAELSNEER	Acetyl:2H(3) (N-term)
		766.0641	2295.1704	3	2294.1066	1.0638	1	1.6	GIVDQSQQAYQEAFEISKK	Acetyl (N-term); 2 Acetyl (K)
		817.994	2450.9602	3	2449.9993	0.9609	0	2.3	DNLTLWTSDTQGDEAEAGEGGEN	Acetyl (N-term)
	(3)41.6	585.7809	1169.5473	2	1168.5738	0.9735	0	0.33	EMQPTHPIR	Oxidation (M); Acetyl:2H(3) (N-term)
		617.8491	1233.6836	2	1233.683	0.0006	0	8.10E-06	DSTLIMQLLR	Acetyl:2H(3) (N-term)
		618.7976	1235.5807	2	1234.5717	1.009	0	0.27	YLAEVAAGDDK	Acetyl (N-term); Acetyl (K)
		625.8468	1249.6791	2	1249.6779	0.0012	0	5.40E-06	DSTLIMQLLR	Oxidation (M); Acetyl:2H(3) (N-term)
		797.3785	1592.7425	2	1592.7357	0.0068	0	2.60E-07	SVTEQGAELSNEER	Acetyl:2H(3) (N-term)

					1009.4381	2016.8616	2	2016.8227	0.039	1	3.3	LAEQAERYDDMAACMK	2 Oxidation (M); Acetyl (N-term); Acetyl (K)
					766.0605	2295.1596	3	2294.1066	1.0529	1	4.5	GIVDQSQQAYQEAFEISKK	Acetyl (N-term); 2 Acetyl (K)
					819.0236	2454.0489	3	2453.0182	1.0307	0	3.5	DNLTLWTSDTQGDEAEAGEGGEN	Acetyl:2H(3) (N-term)
**7**	HSP90AA1	Heat shock protein HSP 90-alpha OS=Homo sapiens OX=9606 GN=HSP90AA1 PE=1 SV=5	107.1±20	(1)21.2	496.2966	990.5786	2	990.5769	0.0016	0	0.021	TKPIWTR	Acetyl:2H(3) (N-term); Acetyl:2H(3) (K)
					640.2934	1278.5721	2	1277.5539	1.0183	0	2.1	DNSTMGYMAAK	Acetyl:2H(3) (N-term); Acetyl:2H(3) (K)
					640.8215	1279.6285	2	1279.6236	0.0049	0	0.00038	DQVANSAFVER	Acetyl:2H(3) (N-term)
					655.8422	1309.6699	2	1308.6654	1.0045	1	0.00032	RAPFDLFENR	Acetyl:2H(3) (N-term)
					662.8026	1323.5905	2	1323.6203	-0.0297	1	2.3	FENLCKIMK	Oxidation (M); Acetyl (N-term); 2 Acetyl (K)
					666.8867	1331.7589	2	1331.7567	0.0022	0	1.3	ADLINNLGTIAK	Acetyl:2H(3) (N-term); Acetyl:2H(3) (K)
					678.8052	1355.5958	2	1355.592	0.0038	0	0.0013	EDQTEYLEER	Acetyl:2H(3) (N-term)

		720.38	1438.7455	2	1438.786	-0.0405	0	2.6	TLTIVDTGIGMTK	Acetyl:2H(3) (N-term); Acetyl:2H(3) (K)
		876.5145	1751.0144	2	1749.9823	1.0321	1	1.2	ADLINNLGTIAKSGTK	Acetyl:2H(3) (N-term); 2 Acetyl:2H(3) (K)
		1239.0741	2476.1336	2	2475.1893	0.9443	0	2.2	VFIMDNCEELIPEYLNFIR	Oxidation (M); Acetyl:2H(3) (N-term)
		877.4069	2629.1988	3	2628.2716	0.9272	1	3	RVFIMDNCEELIPEYLNFIR	Oxidation (M); Acetyl (N-term)
		889.8013	2666.382	3	2666.3242	0.0577	0	3	HGLEVIYMIEPIDEYCVQQLK	Acetyl:2H(3) (N-term); Acetyl:2H(3) (K)
		1358.2291	2714.4436	2	2714.4412	0.0024	1	3.8	TLTIVDTGIGMTKADLINNLGTIAK	Oxidation (M); Acetyl (N-term); 2 Acetyl (K)
		908.8177	2723.4312	3	2723.4977	-0.0665	1	1.1	TLTIVDTGIGMTKADLINNLGTIAK	Oxidation (M); Acetyl:2H(3) (N-term); 2 Acetyl:2H(3) (K)
		1016.8665	3047.5777	3	3046.569	1.0086	0	8.80E-07	DLVILLYETALLSSGFSLEDPQTHANR	Acetyl:2H(3) (N-term)
	(2)12.8	496.3016	990.5887	2	990.5769	0.0117	0	0.054	TKPIWTR	Acetyl:2H(3) (N-term); Acetyl:2H(3) (K)
		640.8269	1279.6392	2	1279.6236	0.0156	0	0.0034	DQVANSAFVER	Acetyl:2H(3) (N-term)
		655.3471	1308.6796	2	1308.6654	0.0142	1	0.001	RAPFDLFENR	Acetyl:2H(3) (N-term)
		666.8917	1331.7689	2	1331.7567	0.0122	0	0.1	ADLINNLGTIAK	Acetyl:2H(3) (N-term); Acetyl:2H(3) (K)

		720.409	1438.8034	2	1438.786	0.0174	0	0.00021	TLTIVDTGIGMTK	Acetyl:2H(3) (N-term); Acetyl:2H(3) (K)
		779.9193	1557.8239	2	1557.8078	0.0162	0	0.00024	GVVDSEDLPLNISR	Acetyl:2H(3) (N-term)
		909.1472	2724.4198	3	2723.4977	0.9221	1	0.78	TLTIVDTGIGMTKADLINNLGTIAK	Oxidation (M); Acetyl:2H(3) (N-term); 2 Acetyl:2H(3) (K)
		1016.5406	3046.6	3	3046.569	0.0309	0	4.40E-08	DLVILLYETALLSSGFSLEDPQTHANR	Acetyl:2H(3) (N-term)
	(3)41.8	496.2963	990.5781	2	990.5769	0.0012	0	0.01	TKPIWTR	Acetyl:2H(3) (N-term); Acetyl:2H(3) (K)
		606.2785	1210.5425	2	1209.5738	0.9687	0	0.0025	LGIHEDSQNR	Acetyl (N-term)
		640.8215	1279.6284	2	1279.6236	0.0048	0	0.00014	DQVANSAFVER	Acetyl:2H(3) (N-term)
		655.3234	1308.6322	2	1307.6254	1.0068	1	1.7	FENLCKIMK	Acetyl (N-term); 2 Acetyl (K)
		655.342	1308.6694	2	1308.6654	0.0041	1	4.70E-05	RAPFDLFENR	Acetyl:2H(3) (N-term)
		656.2996	1310.5846	2	1309.5437	1.0409	0	1.9	DNSTMGYMAAK	2 Oxidation (M); Acetyl:2H(3) (N-term); Acetyl:2H(3) (K)
		657.8477	1313.6808	2	1313.6774	0.0034	0	0.44	HIYYITGETK	Acetyl:2H(3) (N-term); Acetyl:2H(3) (K)
		662.8019	1323.5893	2	1323.6203	-0.031	1	11	FENLCKIMK	Oxidation (M); Acetyl (N-term); 2 Acetyl (K)
		664.3641	1326.7137	2	1325.7191	0.9947	0	3.8	ADLINNLGTIAK	Acetyl (N-term); Acetyl (K)

	666.8869	1331.7592	2	1331.7567	0.0025	0	0.09	ADLINNLGTIAK	Acetyl:2H(3) (N-term); Acetyl:2H(3) (K)
	678.8062	1355.5977	2	1355.592	0.0058	0	1.80E-06	EDQTEYLEER	Acetyl:2H(3) (N-term)
	720.4018	1438.789	2	1438.786	0.003	0	0.25	TLTIVDTGIGMTK	Acetyl:2H(3) (N-term); Acetyl:2H(3) (K)
	779.9135	1557.8125	2	1557.8078	0.0047	0	3.40E-07	GVVDSEDLPLNISR	Acetyl:2H(3) (N-term)
	809.9085	1617.8023	2	1616.7953	1.007	0	0.0021	SLTNDWEDHLAVK	Acetyl:2H(3) (N-term); Acetyl:2H(3) (K)
	761.0615	2280.1628	3	2279.1618	1.001	1	3.1	TLVSVTKEGLELPEDEEEK	Acetyl:2H(3) (N-term); 2 Acetyl:2H(3) (K)
	820.4234	2458.2484	3	2457.2057	1.0427	1	4	GVVDSEDLPLNISREMLQQSK	Oxidation (M); Acetyl (N-term); Acetyl (K)
	1239.0687	2476.1228	2	2475.1893	0.9335	0	5.1	VFIMDNCEELIPEYLNFIR	Oxidation (M); Acetyl:2H(3) (N-term)
	831.3658	2491.0757	3	2490.1421	0.9336	0	1.1	LVTSPCCIVTSTYGWTANMER	Acetyl:2H(3) (N-term)
	877.4095	2629.2066	3	2628.2716	0.935	1	5	RVFIMDNCEELIPEYLNFIR	Oxidation (M); Acetyl (N-term)
	1016.5329	3046.5769	3	3046.569	0.0078	0	1.40E-08	DLVILLYETALLSSGFSLEDPQTHANR	Acetyl:2H(3) (N-term)
	1347.9515	4040.8327	3	4040.7997	0.033	1	3.6	MIKLGLGIDEDDPTADDTSAAVTEEMPPLEGDDDTSR	2 Oxidation (M); Acetyl:2H(3) (N-term); Acetyl:2H(3) (K)

					1086.5501	5427.7141	5	5426.6008	1.1133	1	0.98	PEETQTQDQPMEEEEVETFAFQAEIAQLMSLIINTFYSNKEIFLR	Acetyl:2H(3) (N-term); Acetyl:2H(3) (K)
**8**	EEF1D	Elongation factor 1-delta OS=Homo sapiens OX=9606 GN=EEF1D PE=1 SV=5	106.4±8.7 (n=2)	(1)17.4	396.2414	1185.7023	3	1184.6917	1.0106	1	8	LVPVGYGIRK	Acetyl (N-term); Acetyl (K)
					702.3829	1402.7512	2	1402.7495	0.0017	0	2.10E-07	IASLEVENQSLR	Acetyl:2H(3) (N-term)
					742.8729	1483.7313	2	1483.7281	0.0033	0	0.067	ATAPQTQHVSPMR	Oxidation (M); Acetyl:2H(3) (N-term)
					794.3674	1586.7203	2	1586.7227	-0.0023	0	0.023	FYEQMNGPVAGASR	Oxidation (M); Acetyl:2H(3) (N-term)
				(3)23.8	702.3846	1402.7547	2	1402.7495	0.0052	0	2.30E-06	IASLEVENQSLR	Acetyl:2H(3) (N-term)
					734.8755	1467.7365	2	1467.7332	0.0033	0	0.043	ATAPQTQHVSPMR	Acetyl:2H(3) (N-term)
					786.3758	1570.737	2	1570.7277	0.0093	0	0.00013	FYEQMNGPVAGASR	Acetyl:2H(3) (N-term)
					1014.5165	2027.0184	2	2027.0173	0.0012	1	1.9	MATNFLAHEKIWFDK	Acetyl (Protein N-term); Acetyl:2H(3) (N-term); 2 Acetyl:2H(3) (K)
					1052.5342	2103.0538	2	2102.0076	1.0463	1	2.7	SSPGHRATAPQTQHVSPMR	Oxidation (M); Acetyl (N-term)

					1137.5504	2273.0862	2	2272.127	0.9592	1	2.2	ATAPQTQHVSP MRQVEPPAK	Oxidation (M); Acetyl (N-term); Acetyl (K)
**9**	NPM1	Nucleophosmin OS=Homo sapiens OX=9606 GN=NPM1 PE=1 SV=2	71.21±8.3	(1)33	415.2554	828.4963	2	828.4957	0.0007	0	1.2	VTLATLK	Acetyl (N-term); Acetyl (K)
					489.7779	977.5413	2	976.546	0.9953	1	0.4	SIRDTPAK	Acetyl:2H(3) (N-term); Acetyl:2H(3) (K)
					556.3038	1110.5931	2	1110.5921	0.001	1	2	GGSLPKVEAK	Acetyl (N-term); 2 Acetyl (K)
					807.3847	1612.7549	2	1612.752	0.0029	0	3.40E-09	VDNDENEHQLS LR	Acetyl:2H(3) (N-term)
					940.9441	1879.8736	2	1879.8602	0.0134	0	7.20E-10	MTDQEAIQDL WQWR	Oxidation (M); Acetyl:2H(3) (N-term)
					758.4213	2272.242	3	2271.2376	1.0043	0	0.061	MSVQPTVSLGG FEITPPVVLR	Acetyl:2H(3) (N-term)
					763.4191	2287.2355	3	2287.2326	0.0029	0	6.30E-08	MSVQPTVSLGG FEITPPVVLR	Oxidation (M); Acetyl:2H(3) (N-term)
					999.7823	2996.3252	3	2996.3005	0.0247	0	1.5	MEDSMDMDMS PLRPQNYLFGC ELK	Acetyl:2H(3) (N-term); Acetyl:2H(3) (K)
					765.9531	3059.7834	4	3058.7259	1.0575	1	3.8	VTLATLKMSVQ PTVSLGGFEITP PVVLR	Oxidation (M); Acetyl:2H(3) (N-term); Acetyl:2H(3) (K)

	(2)34.7	404.2623	806.5101	2	806.502	0.0081	0	6.30E-05	LLSISGK	Acetyl:2H(3) (N-term); Acetyl:2H(3) (K)
		418.2783	834.5421	2	834.5333	0.0088	0	0.38	VTLATLK	Acetyl:2H(3) (N-term); Acetyl:2H(3) (K)
		482.3115	962.6085	2	962.6031	0.0053	1	3	LLSISGKR	Acetyl:2H(3) (N-term); Acetyl:2H(3) (K)
		489.782	977.5494	2	976.546	1.0034	1	1.1	SIRDTPAK	Acetyl:2H(3) (N-term); Acetyl:2H(3) (K)
		807.3914	1612.7683	2	1612.752	0.0163	0	0.0013	VDNDENEHQLS LR	Acetyl:2H(3) (N-term)
		940.9481	1879.8817	2	1879.8602	0.0215	0	6.10E-10	MTDQEAIQDL WQWR	Oxidation (M); Acetyl:2H(3) (N-term)
		758.0951	2271.2634	3	2271.2376	0.0257	0	3.70E-07	MSVQPTVSLGG FEITPPVVLR	Acetyl:2H(3) (N-term)
		763.4268	2287.2586	3	2287.2326	0.026	0	1.40E-07	MSVQPTVSLGG FEITPPVVLR	Oxidation (M); Acetyl:2H(3) (N-term)
		1145.156	2288.2974	2	2287.2326	1.0649	0	0.21	MSVQPTVSLGG FEITPPVVLR	Oxidation (M); Acetyl:2H(3) (N-term)
		1157.8832	3470.6278	3	3469.5222	1.1056	0	1.2	CGSGPVHISGQ HLVAVEEDAES EDEEEEDVK	Acetyl:2H(3) (N-term); Acetyl:2H(3) (K)
	(3)36.7	418.2751	834.5355	2	834.5333	0.0022	0	1.2	VTLATLK	Acetyl:2H(3) (N-term); Acetyl:2H(3) (K)
		489.7768	977.5391	2	976.546	0.9931	1	0.79	SIRDTPAK	Acetyl:2H(3) (N-term); Acetyl:2H(3) (K)

					532.7793	1063.5439	2	1063.5417	0.0023	0	1.1	DSKPSSTPR	Acetyl:2H(3) (N-term); Acetyl:2H(3) (K)
					1147.5541	1146.5468	1	1146.5591	-0.0122	1	1	AKMQASIEK	Oxidation (M); Acetyl (N-term); 2 Acetyl (K)
					807.3887	1612.7628	2	1612.752	0.0108	0	1.60E-08	VDNDENEHQLS LR	Acetyl:2H(3) (N-term)
					940.94	1879.8655	2	1879.8602	0.0053	0	2.10E-07	MTDQEAIQDL WQWR	Oxidation (M); Acetyl:2H(3) (N-term)
					1136.626	2271.2374	2	2271.2376	-0.0002	0	1.90E-12	MSVQPTVSLGG FEITPPVVLR	Acetyl:2H(3) (N-term)
					758.0874	2271.2404	3	2271.2376	0.0028	0	3.90E-08	MSVQPTVSLGG FEITPPVVLR	Acetyl:2H(3) (N-term)
					763.4195	2287.2367	3	2287.2326	0.0041	0	3.10E-09	MSVQPTVSLGG FEITPPVVLR	Oxidation (M); Acetyl:2H(3) (N-term)
					1145.1429	2288.2712	2	2287.2326	1.0387	0	0.0079	MSVQPTVSLGG FEITPPVVLR	Oxidation (M); Acetyl:2H(3) (N-term)
					1629.6906	3257.3666	2	3256.4383	0.9284	1	1.9	EDSMDMDMSP LRPQNYLFGCE LKADK	2 Oxidation (M); Acetyl:2H(3) (N-term); 2 Acetyl:2H(3) (K)
					850.6523	3398.5802	4	3397.4995	1.0807	1	1.8	MEDSMDMDMS PLRPQNYLFGC ELKADK	Acetyl (Protein N-term); Acetyl:2H(3) (N-term); 2 Acetyl:2H(3) (K)
**10**	ATP5F 1B	ATP synthase subunit beta, mitochondrial OS=Homo	56.38±32	(1)36.5	530.7918	1059.569	2	1058.5761	0.9929	0	0.71	IGLFGGAGVGK	Acetyl (N-term); Acetyl (K)

	sapiens OX=9606 GN=ATP5F1B PE=1 SV=3									
		723.8702	1445.7258	2	1445.7263	-0.0005	0	0.01	IMNVIGEPIDER	Oxidation (M); Acetyl:2H(3) (N-term)
		742.9137	1483.8129	2	1483.8114	0.0015	0	1.70E-09	VALTGLTVAEYFR	Acetyl:2H(3) (N-term)
		832.4243	1662.834	2	1661.8275	1.0065	0	0.35	VALVYGQMNEPPGAR	Oxidation (M); Acetyl:2H(3) (N-term)
		938.9526	1875.8906	2	1875.8864	0.0042	0	1.20E-05	IMDPNIVGSEHYDVAR	Oxidation (M); Acetyl:2H(3) (N-term)
		1017.5372	2033.0598	2	2032.0556	1.0043	0	1.60E-05	AIAELGIYPAVDPLDSTSR	Acetyl:2H(3) (N-term)
		1164.0593	2326.104	2	2326.1013	0.0028	0	2.7	IPSAVGYQPTLATDMGTMQER	Oxidation (M); Acetyl:2H(3) (N-term)
		1355.2145	2708.4144	2	2707.4758	0.9386	1	3.1	FLSQPFQVAEVFTGHMGKLVPLK	Acetyl:2H(3) (N-term); 2 Acetyl:2H(3) (K)
		1358.2291	2714.4436	2	2714.4142	0.0294	1	0.96	FLSQPFQVAEVFTGHMGKLVPLK	Oxidation (M); Acetyl (N-term); 2 Acetyl (K)
		1026.2449	3075.7129	3	3075.7048	0.0081	0	6.00E-09	IVAVIGAVVDVQFDEGLPPILNALEVQGR	Acetyl:2H(3) (N-term)

	1050.838	3149.4922	3	3149.5572	-0.065	0	0.36	QFAPIHAEAPEFMEMSVEQEILVTGIK	Oxidation (M); Acetyl:2H(3) (N-term); Acetyl:2H(3) (K)
	1051.1654	3150.4744	3	3149.5572	0.9172	0	0.64	QFAPIHAEAPEFMEMSVEQEILVTGIK	Oxidation (M); Acetyl:2H(3) (N-term); Acetyl:2H(3) (K)
	1220.353	3658.0372	3	3656.989	1.0482	1	0.76	AGAATGRIVAVIGAVVDVQFDEGLPPILNALEVQGR	Acetyl (N-term)
(2)50.7	530.7975	1059.5804	2	1058.5761	1.0044	0	3.5	IGLFGGAGVGK	Acetyl (N-term); Acetyl (K)
	628.2933	1254.5721	2	1253.6046	0.9675	0	4.2	DYAAQTSPSPK	Acetyl:2H(3) (N-term); Acetyl:2H(3) (K)
	660.8088	1319.6031	2	1319.6391	-0.036	0	5.7	TIAMDGTEGLVR	Oxidation (M); Acetyl (N-term)
	662.3443	1322.674	2	1322.6579	0.0161	0	0.021	TIAMDGTEGLVR	Oxidation (M); Acetyl:2H(3) (N-term)
	724.3805	1446.7465	2	1445.7263	1.0202	0	0.1	IMNVIGEPIDER	Oxidation (M); Acetyl:2H(3) (N-term)
	740.9032	1479.7919	2	1479.7761	0.0158	0	5.90E-08	FTQAGSEVSALLGR	Acetyl:2H(3) (N-term)
	742.9208	1483.827	2	1483.8114	0.0156	0	6.20E-08	VALTGLTVAEYFR	Acetyl:2H(3) (N-term)
	809.9891	1617.9636	2	1617.9454	0.0182	0	1.1	LTPSASLPPAQLLLR	Acetyl (N-term)

	823.9335	1645.8524	2	1645.8325	0.0199	0	0.0023	VALVYGQMNEPPGAR	Acetyl:2H(3) (N-term)
	831.9343	1661.854	2	1661.8275	0.0265	0	0.59	VALVYGQMNEPPGAR	Oxidation (M); Acetyl:2H(3) (N-term)
	853.4211	1704.8276	2	1704.8332	-0.0056	1	1.3	AHGGYSVFAGVGERTR	Acetyl (N-term)
	984.0119	1966.0092	2	1965.9875	0.0217	0	4.10E-12	DQEGQDVLLFIDNIFR	Acetyl:2H(3) (N-term)
	1057.5448	2113.075	2	2112.0621	1.0129	0	0.011	FLSQPFQVAEVFTGHMGK	Acetyl:2H(3) (N-term); Acetyl:2H(3) (K)
	782.0498	2343.1276	3	2342.0962	1.0314	0	0.87	IPSAVGYQPTLATDMGTMQER	2 Oxidation (M); Acetyl:2H(3) (N-term)
	1354.714	2707.4134	2	2707.4758	-0.0624	1	0.73	FLSQPFQVAEVFTGHMGKLVPLK	Acetyl:2H(3) (N-term); 2 Acetyl:2H(3) (K)
	1026.2543	3075.7411	3	3075.7048	0.0363	0	7.40E-10	IVAVIGAVVDVQFDEGLPPILNALEVQGR	Acetyl:2H(3) (N-term)
	1050.8449	3149.5129	3	3149.5572	-0.0443	0	1.6	QFAPIHAEAPEFMEMSVEQEILVTGIK	Oxidation (M); Acetyl:2H(3) (N-term); Acetyl:2H(3) (K)
	788.3858	3149.5139	4	3149.5572	-0.0433	0	2.1	QFAPIHAEAPEFMEMSVEQEILVTGIK	Oxidation (M); Acetyl:2H(3) (N-term); Acetyl:2H(3) (K)

		1050.8455	3149.5147	3	3149.5572	-0.0425	0	0.75	QFAPIHAEAPEF MEMSVEQEILV TGIK	Oxidation (M); Acetyl:2H(3) (N-term); Acetyl:2H(3) (K)
		1272.6485	3814.9237	3	3814.875	0.0487	1	1.4	GFQQILAGEYD HLPEQAFYMVG PIEEAVAKADK	Oxidation (M); Acetyl:2H(3) (N-term); 2 Acetyl:2H(3) (K)
	(3)46.1	513.2788	1024.5431	2	1024.5665	-0.0234	0	1.2	VAAAPASGALR	Acetyl (N-term)
		533.8086	1065.6027	2	1064.6137	0.989	0	2	IGLFGGAGVGK	Acetyl:2H(3) (N-term); Acetyl:2H(3) (K)
		542.3165	1082.6185	2	1082.6163	0.0022	0	0.084	IPVGPETLGR	Acetyl:2H(3) (N-term)
		663.8962	1325.7779	2	1325.7541	0.0238	1	1.9	GVQKILQDYK	Acetyl:2H(3) (N-term); 2 Acetyl:2H(3) (K)
		714.8496	1427.6845	2	1426.7126	0.972	0	3.6	IMNVIGEPIDER	Acetyl (N-term)
		740.8972	1479.7798	2	1479.7761	0.0037	0	8.00E-09	FTQAGSEVSAL LGR	Acetyl:2H(3) (N-term)
		742.9142	1483.8139	2	1483.8114	0.0025	0	7.20E-10	VALTGLTVAEY FR	Acetyl:2H(3) (N-term)
		774.4539	1546.8933	2	1546.8911	0.0022	0	0.00023	TVLIMELINNV AK	Acetyl:2H(3) (N-term); Acetyl:2H(3) (K)
		831.9229	1661.8311	2	1661.8275	0.0037	0	0.61	VALVYGQMNE PPGAR	Oxidation (M); Acetyl:2H(3) (N-term)
		984.0023	1965.9901	2	1965.9875	0.0026	0	8.30E-10	DQEGQDVLLFI DNIFR	Acetyl:2H(3) (N-term)
		1057.0407	2112.0668	2	2112.0621	0.0047	0	2.40E-05	FLSQPFQVAEV FTGHMGK	Acetyl:2H(3) (N-term); Acetyl:2H(3) (K)

					1113.0679	2224.1212	2	2224.0939	0.0274	0	1.1	SLQDIIAILGMD ELSEEDK	Oxidation (M); Acetyl:2H(3) (N-term); Acetyl:2H(3) (K)
					771.0439	2310.1098	3	2310.1063	0.0035	0	1.00E-05	IPSAVGYQPTLA TDMGTMQER	Acetyl:2H(3) (N-term)
					1156.5543	2311.094	2	2310.1063	0.9877	0	7.70E-08	IPSAVGYQPTLA TDMGTMQER	Acetyl:2H(3) (N-term)
					1164.0551	2326.0956	2	2326.1013	-0.0056	0	2.2	IPSAVGYQPTLA TDMGTMQER	Oxidation (M); Acetyl:2H(3) (N-term)
					1355.2091	2708.4036	2	2707.4758	0.9278	1	3.2	FLSQPFQVAEV FTGHMGKLVPL K	Acetyl:2H(3) (N-term); 2 Acetyl:2H(3) (K)
					1026.2446	3075.712	3	3075.7048	0.0072	0	4.90E-10	IVAVIGAVVDV QFDEGLPPILNA LEVQGR	Acetyl:2H(3) (N-term)
					1050.8327	3149.4763	3	3149.5572	-0.0809	0	1.2	QFAPIHAEAPEF MEMSVEQEILV TGIK	Oxidation (M); Acetyl:2H(3) (N-term); Acetyl:2H(3) (K)
**11**	ACTN 4	Alpha-actinin-4 OS=Homo sapiens OX=9606 GN=ACTN4 PE=1 SV=2	38.59±12 (n=2)	(2)22	488.2871	974.5596	2	973.5286	1.0311	1	2.5	EAMLKHR	Acetyl:2H(3) (N-term); Acetyl:2H(3) (K)
					527.2575	1052.5004	2	1052.4999	0.0005	0	3.1	QQSNEHLR	Acetyl (N-term)

716.4129	1430.8112	2	1430.7961	0.0152	0	1.10E-06	VGWEQLLTTIA R	Acetyl:2H(3) (N-term)
626.3029	1875.8869	3	1875.8713	0.0156	0	2.5	MAPYQGPDAV PGALDYK	Acetyl (N-term); Acetyl (K)
754	2258.9782	3	2258.0208	0.9573	1	0.79	CQKICDQWDAL GSLTHSR	Acetyl (N-term); Acetyl (K)
948.5508	2842.6307	3	2841.5428	1.0879	1	2.4	VGWEQLLTTIA RTINEVENQILT R	Acetyl:2H(3) (N-term)
984.157	2949.4492	3	2949.4183	0.0309	0	7.00E-07	VEQIAAIAQELN ELDYYDSHNVN TR	Acetyl:2H(3) (N-term)
1020.5432	3058.6078	3	3058.6083	-0.0005	1	1.1	LVSIGAEEIVDG NAKMTLGMIW TIILR	2 Oxidation (M); Acetyl (N-term); Acetyl (K)
1316.6401	3946.8985	3	3946.8393	0.0591	1	2.5	MLDAEDIVNTA RPDEKAIMTYV SSFYHAFSGAQ K	Oxidation (M); Acetyl (N-term); 2 Acetyl (K)
1420.2384	4257.6934	3	4256.6807	1.0127	0	0.97	MVDYHAANQS YQYGPSSAGNG AGGGGSMGDY MAQEDDWDR	Acetyl (N-term)
1426.2367	4275.6883	3	4275.6944	-0.0062	0	0.72	MVDYHAANQS YQYGPSSAGNG	Oxidation (M); Acetyl:2H(3) (N-term)

									AGGGGSMGDY MAQEDDWDR	
		1439.2651	4314.7735	3	4314.6862	0.0873	0	0.96	MVDYHAANQS YQYGPSSAGNG AGGGGSMGDY MAQEDDWDR	Acetyl (Protein N-term); Oxidation (M); Acetyl (N-term)
		1451.2645	4350.7717	3	4349.6949	1.0768	0	0.71	MVDYHAANQS YQYGPSSAGNG AGGGGSMGDY MAQEDDWDR	Acetyl (Protein N-term); 3 Oxidation (M); Acetyl:2H(3) (N-term)
	(3)15.1	527.2534	1052.4922	2	1052.4999	-0.0077	0	2.1	QQSNEHLR	Acetyl (N-term)
		528.7685	1055.5224	2	1055.5187	0.0036	0	0.01	QQSNEHLR	Acetyl:2H(3) (N-term)
		569.3024	1136.5903	2	1136.5905	-0.0002	0	0.0047	NFITAEELR	Acetyl:2H(3) (N-term)
		602.8045	1203.5944	2	1203.5923	0.0021	1	0.93	STLPDADRER	Acetyl:2H(3) (N-term)
		627.2935	1252.5724	2	1252.5935	-0.0212	0	3.1	DHALLEEQSK	Acetyl (N-term); Acetyl (K)
		630.8556	1259.6966	2	1259.6953	0.0013	0	8.90E-05	LASDLLEWIR	Acetyl:2H(3) (N-term)
		635.3893	1268.7641	2	1268.7359	0.0282	1	1.1	EREAILAIHK	Acetyl:2H(3) (N-term); Acetyl:2H(3) (K)
		709.4124	1416.8102	2	1416.8089	0.0013	0	0.00059	LMLLLEVISGER	Acetyl:2H(3) (N-term)
		716.4067	1430.7988	2	1430.7961	0.0027	0	8.60E-07	VGWEQLLTTIA R	Acetyl:2H(3) (N-term)
		717.4092	1432.8038	2	1432.8038	-0.0001	0	7.10E-05	LMLLLEVISGER	Oxidation (M); Acetyl:2H(3) (N-term)

					984.149	2949.4252	3	2949.4183	0.0069	0	6.70E-07	VEQIAAIAQELNELDYYDSHNVNTR	Acetyl:2H(3) (N-term)
					1322.3026	3963.886	3	3962.8343	1.0517	1	1.2	MLDAEDIVNTARPDEKAIMTYVSSFYHAFSGAQK	2 Oxidation (M); Acetyl (N-term); 2 Acetyl (K)
**12**	ACTG 1	Actin, cytoplasmic 2 OS=Homo sapiens OX=9606 GN=ACTG1 PE=1 SV=1	47.8±16 (n=2)	(1)21.6	511.2426	1020.4706	2	1020.4704	0.0002	0	5.40E-05	AGFAGDDAPR	Acetyl:2H(3) (N-term)
					589.2833	1176.552	2	1176.549	0.0029	0	3.70E-05	GYSFTTTAER	Acetyl:2H(3) (N-term)
					781.3731	1560.7315	2	1560.7248	0.0068	0	0.0069	QEYDESGPSIVHR	Acetyl:2H(3) (N-term)
					661.9649	1982.8728	3	1982.9084	-0.0356	0	1.8	EEEIAALVIDNGSGMCK	Acetyl (Protein N-term); Oxidation (M); Acetyl:2H(3) (N-term); Acetyl:2H(3) (K)
					1008.797	3023.3692	3	3023.3311	0.0381	1	1.1	MEEEIAALVIDNGSGMCKAGFAGDDAPR	Oxidation (M); Acetyl (N-term); Acetyl (K)

		1028.4437	3082.3093	3	3081.3365	0.9727	1	1.2	MEEEIAALVIDNGSGMCKAGFAGDDAPR	Acetyl (Protein N-term); 2 Oxidation (M); Acetyl (N-term); Acetyl (K)
		1076.8763	3227.6071	3	3227.6364	-0.0294	0	1.5	TTGIVMDSGDGVTHTVPIYEGYALPHAILR	Acetyl:2H(3) (N-term)
	(3)44.3	419.2407	836.4669	2	836.4756	-0.0087	0	0.72	IIAPPER	Acetyl (N-term)
		511.2433	1020.472	2	1020.4704	0.0016	0	1.10E-05	AGFAGDDAPR	Acetyl:2H(3) (N-term)
		589.2837	1176.5529	2	1176.549	0.0039	0	1.20E-05	GYSFTTTAER	Acetyl:2H(3) (N-term)
		513.305	1536.8931	3	1536.8783	0.0148	1	1.6	EITALAPSTMKIK	Acetyl:2H(3) (N-term); 2 Acetyl:2H(3) (K)
		781.3715	1560.7284	2	1560.7248	0.0036	0	1.80E-08	QEYDESGPSIVHR	Acetyl:2H(3) (N-term)
		837.9277	1673.8408	2	1673.8452	-0.0044	1	3.2	GYSFTTTAEREIVR	Acetyl:2H(3) (N-term)
		918.4671	1834.9196	2	1834.914	0.0056	0	0.0021	SYELPDGQVITIGNER	Acetyl:2H(3) (N-term)
		677.6276	2029.8609	3	2029.9091	-0.0483	0	1.9	YPIEHGIVTNWDDMEK	Acetyl (N-term); Acetyl (K)
		1028.4451	3082.3135	3	3081.3365	0.9769	1	1.9	MEEEIAALVIDNGSGMCKAGFAGDDAPR	Acetyl (Protein N-term); 2 Oxidation (M); Acetyl (N-term); Acetyl (K)
		1030.4872	3088.4398	3	3087.3742	1.0656	1	1.2	MEEEIAALVIDNGSGMCKAGFAGDDAPR	Acetyl (Protein N-term); 2 Oxidation (M); Acetyl:2H(3) (N-term); Acetyl:2H(3) (K)

					1077.2232	3228.6478	3	3227.6364	1.0113	0	3.30E-06	TTGIVMDSGDG VTHTVPIYEGY ALPHAILR	Acetyl:2H(3) (N-term)
					1116.5455	3346.6147	3	3345.6163	0.9984	0	0.77	MTQIMFETFNT PAMYVAIQAVL SLYASGR	3 Oxidation (M); Acetyl:2H(3) (N-term)
**13**	NASP	Nuclear autoantigenic sperm protein OS=Homo sapiens OX=9606 GN=NASP PE=1 SV=2	27.05±12 (n=2)	(1)19.8	423.761	845.5074	2	845.505	0.0025	0	0.046	SLLELAR	Acetyl:2H(3) (N-term)
					546.3156	1090.6165	2	1090.6141	0.0025	0	8.2	SGNVAELALK	Acetyl:2H(3) (N-term); Acetyl:2H(3) (K)
					716.8607	1431.7068	2	1431.7033	0.0035	0	1.80E-06	VQIAANEETQE R	Acetyl:2H(3) (N-term)
					968.4822	1934.9498	2	1934.9143	0.0355	0	0.87	AMESTATAAV AAELVSADK	Oxidation (M); Acetyl (N-term); Acetyl (K)
					1125.4575	2248.9004	2	2248.8999	0.0006	1	1.1	EEQMKEGEETE GSEEDDK	Oxidation (M); Acetyl:2H(3) (N-term); 2 Acetyl:2H(3) (K)
					835.4083	2503.203	3	2503.194	0.009	0	4.40E-07	ATLVESSTSGFT PGGGGSSVSMI ASR	Oxidation (M); Acetyl:2H(3) (N-term)

	1086.5244	3256.5514	3	3256.604	-0.0526	1	1	SLLELARMENG VLGNALEGVH VEEEEGEK	Oxidation (M); Acetyl:2H(3) (N-term); Acetyl:2H(3) (K)
	1090.5359	3268.5859	3	3268.5835	0.0024	0	6.00E-07	LLAETHYQLGL AYGYNSQYDE AVAQFSK	Acetyl:2H(3) (N-term); Acetyl:2H(3) (K)
	1157.877	3470.6092	3	3469.6678	0.9414	1	1.3	MAMESTATAA VAAELVSADKI EDVPAPSTSAD K	Acetyl (Protein N-term); Oxidation (M); Acetyl:2H(3) (N-term); 2 Acetyl:2H(3) (K)
(3)30.7	423.7616	845.5085	2	845.505	0.0036	0	0.011	SLLELAR	Acetyl:2H(3) (N-term)
	678.3416	1354.6687	2	1354.698	-0.0293	1	1.1	AKLVPSQEETK	Acetyl (N-term); 2 Acetyl (K)
	716.8616	1431.7086	2	1431.7033	0.0053	0	4.50E-06	VQIAANEETQE R	Acetyl:2H(3) (N-term)
	716.8912	1431.7679	2	1431.7628	0.005	0	0.0011	EAQLYAAQAH LK	Acetyl:2H(3) (N-term); Acetyl:2H(3) (K)
	803.8554	1605.6963	2	1604.6876	1.0087	0	3.9	TEEMPNDSVLE NK	Oxidation (M); Acetyl (N-term); Acetyl (K)
	971.0091	1940.0036	2	1938.9581	1.0456	0	1.1	VDLTLDWLTET SEEAK	Acetyl:2H(3) (N-term); Acetyl:2H(3) (K)
	971.9884	1941.9622	2	1940.9519	1.0103	0	2.3	AMESTATAAV AAELVSADK	Oxidation (M); Acetyl:2H(3) (N-term); Acetyl:2H(3) (K)
	662.324	1983.9503	3	1982.9625	0.9878	0	2.7	AMESTATAAV AAELVSADK	Acetyl (Protein N-term); Oxidation (M);

									Acetyl:2H(3) (N-term); Acetyl:2H(3) (K)
	1037.5002	2072.9858	2	2071.9924	0.9934	0	1.4	MAMESTATAAVAAELVSADK	Oxidation (M); Acetyl:2H(3) (N-term); Acetyl:2H(3) (K)
	1055.0129	2108.0112	2	2107.9653	0.0459	0	0.21	MAMESTATAAVAAELVSADK	Acetyl (Protein N-term); Oxidation (M); Acetyl (N-term); Acetyl (K)
	743.6793	2228.016	3	2227.0539	0.9621	0	1.5	KPTDGASSSNCVTDISHLVR	Acetyl (N-term); Acetyl (K)
	745.3544	2233.0415	3	2233.077	-0.0355	1	1.8	MAVLNEQVKEAEGSSAEYK	Oxidation (M); Acetyl:2H(3) (N-term); 2 Acetyl:2H(3) (K)
	1218.6359	2435.2572	2	2435.2519	0.0054	0	0.91	HLVMGDIPAAVNAFQEAASLLGK	Acetyl (N-term); Acetyl (K)
	1238.0836	2474.1526	2	2474.1389	0.0137	0	0.3	MENGVLGNALEGVHVEEEEGEK	Oxidation (M); Acetyl:2H(3) (N-term); Acetyl:2H(3) (K)
	830.074	2487.2003	3	2487.1991	0.0012	0	1.90E-05	ATLVESSTSGFTPGGGGSSVSMIASR	Acetyl:2H(3) (N-term)
	835.743	2504.2073	3	2503.194	1.0133	0	0.13	ATLVESSTSGFTPGGGGSSVSMIASR	Oxidation (M); Acetyl:2H(3) (N-term)

					1090.5384	3268.5934	3	3268.5835	0.0099	0	3.40E-08	LLAETHYQLGL AYGYNSQYDE AVAQFSK	Acetyl:2H(3) (N-term); Acetyl:2H(3) (K)
					1157.8749	3470.6029	3	3469.6678	0.9351	1	0.78	MAMESTATAA VAAELVSADKI EDVPAPSTSAD K	Acetyl (Protein N-term); Oxidation (M); Acetyl:2H(3) (N-term); 2 Acetyl:2H(3) (K)
**14**	PRDX 4	Peroxiredoxin -4 OS=Homo sapiens OX=9606 GN=PRDX4 PE=1 SV=1	18.54±5.2 (n=2)	(1)15.9	777.9556	1553.8967	2	1553.8936	0.0032	0	3.00E-06	IPLLSDLTHQIS K	Acetyl:2H(3) (N-term); Acetyl:2H(3) (K)
					835.9031	1669.7917	2	1668.7823	1.0094	0	0.00068	DYGVYLEDSGH TLR	Acetyl:2H(3) (N-term)
					890.9459	1779.8772	2	1778.9064	0.9707	0	0.95	MEALPLLAATT PDHGR	Acetyl (Protein N-term); Acetyl:2H(3) (N-term)
				(3)26.6	777.9558	1553.8971	2	1553.8936	0.0036	0	6.10E-06	IPLLSDLTHQIS K	Acetyl:2H(3) (N-term); Acetyl:2H(3) (K)
					835.4029	1668.7913	2	1668.7823	0.009	0	2.90E-05	DYGVYLEDSGH TLR	Acetyl:2H(3) (N-term)
					951.031	1900.0474	2	1899.0154	1.0321	1	6.1	EALPLLAATTP DHGRHR	Acetyl:2H(3) (N-term)
					1014.5049	2026.9952	2	2027.037	-0.0418	1	3	MEALPLLAATT PDHGRHR	Acetyl (N-term)

	1116.2289	3345.6649	3	3345.6461	0.0188	1	1	GKYLVFFFYPL DFTFVCPTEIIAF GDR	Acetyl (N-term); Acetyl (K)

Table S2. Core signaling pathways with which the target proteins of baicalin are associated.

Ingenuity Canonical Pathways	-log(p-value)	Molecules (gene names)
**Role of PKR in Interferon Induction and Antiviral Response**	5.84	*HSP90AA1,HSP90AB1,HSPA1A/HSPA1B,NPM1*
**PI3K/AKT Signaling**	5.15	*HSP90AA1,HSP90AB1,YWHAQ,YWHAZ*
**eNOS Signaling**	3.89	*HSP90AA1,HSP90AB1,HSPA1A/HSPA1B*
**Aldosterone Signaling in Epithelial Cells**	3.83	*HSP90AA1,HSP90AB1,HSPA1A/HSPA1B*
**NRF2-mediated Oxidative Stress Response**	3.47	*ACTG1,HSP90AA1,HSP90AB1*
**Glucocorticoid Receptor Signaling**	3.45	*ATP5F1B,HSP90AA1,HSP90AB1, HSPA1A/HSPA1B*
**Cell Cycle: G2/M DNA Damage Checkpoint Regulation**	3.3	*YWHAQ,YWHAZ*
**Protein Ubiquitination Pathway**	3.16	*HSP90AA1,HSP90AB1,HSPA1A/HSPA1B*
**Mitotic Roles of Polo-Like Kinase**	3.1	*HSP90AA1,HSP90AB1*
**Remodeling of Epithelial Adherens Junctions**	3.06	*ACTG1,ACTN4*
**ERK5 Signaling**	2.99	*YWHAQ,YWHAZ*
**Hypoxia Signaling in the Cardiovascular System**	2.99	*HSP90AA1,HSP90AB1*
**Xenobiotic Metabolism AHR Signaling Pathway**	2.91	*HSP90AA1,HSP90AB1*

**BAG2 Signaling Pathway**	2.89	*HSP90AA1,HSPA1A/HSPA1B*
**HIPPO signaling**	2.85	*YWHAQ,YWHAZ*
**VEGF Signaling**	2.73	*ACTG1,ACTN4*
**Estrogen Receptor Signaling**	2.68	*ATP5F1B,HSP90AA1,HSP90AB1*
**Telomerase Signaling**	2.67	*HSP90AA1,HSP90AB1*
**PPAR Signaling**	2.67	*HSP90AA1,HSP90AB1*
**IGF-1 Signaling**	2.67	*YWHAQ,YWHAZ*
**Paxillin Signaling**	2.65	*ACTG1,ACTN4*
**Prostate Cancer Signaling**	2.63	*HSP90AA1,HSP90AB1*
**Nitric Oxide Signaling in the Cardiovascular System**	2.59	*HSP90AA1,HSP90AB1*
**Neuregulin Signaling**	2.59	*HSP90AA1,HSP90AB1*
**14-3-3-mediated Signaling**	2.51	*YWHAQ,YWHAZ*
**p70S6K Signaling**	2.49	*YWHAQ,YWHAZ*
**Creatine-phosphate Biosynthesis**	2.48	*CKB*
**MSP-RON Signaling In Cancer Cells Pathway**	2.44	*YWHAQ,YWHAZ*
**Aryl Hydrocarbon Receptor Signaling**	2.38	*HSP90AA1,HSP90AB1*
**Epithelial Adherens Junction Signaling**	2.33	*ACTG1,ACTN4*
**Inhibition of ARE-Mediated mRNA Degradation Pathway**	2.31	*YWHAQ,YWHAZ*
**Germ Cell-Sertoli Cell Junction Signaling**	2.27	*ACTG1,ACTN4*
**Xenobiotic Metabolism CAR Signaling Pathway**	2.25	*HSP90AA1,HSP90AB1*
**Xenobiotic Metabolism PXR**	2.22	*HSP90AA1,HSP90AB1*

**Signaling Pathway**		
**IL-17 Signaling**	2.21	*HSP90AA1,HSP90AB1*
**PPARα/RXRα Activation**	2.2	*HSP90AA1,HSP90AB1*
**Leukocyte Extravasation Signaling**	2.16	*ACTG1,ACTN4*
**ILK Signaling**	2.15	*ACTG1,ACTN4*
**Sertoli Cell-Sertoli Cell Junction Signaling**	2.11	*ACTG1,ACTN4*
**HIF1α Signaling**	2.1	*HSP90AA1,HSPA1A/HSPA1B*
**Integrin Signaling**	2.1	*ACTG1,ACTN4*
**ERK/MAPK Signaling**	2.06	*YWHAQ,YWHAZ*
**Actin Cytoskeleton Signaling**	1.96	*ACTG1,ACTN4*
**Th17 Activation Pathway**	1.9	*HSP90AA1,HSP90AB1*
**Xenobiotic Metabolism Signaling**	1.86	*HSP90AA1,HSP90AB1*
**Huntington's Disease Signaling**	1.85	*ATP5F1B,HSPA1A/HSPA1B*
**Role of p14/p19ARF in Tumor Suppression**	1.73	*NPM1*
**Complement System**	1.62	*C1QBP*
**Protein Kinase A Signaling**	1.57	*YWHAQ,YWHAZ*
**Mechanisms of Viral Exit from Host Cells**	1.57	*ACTG1*
**MSP-RON Signaling Pathway**	1.42	*ACTG1*
**Agrin Interactions at Neuromuscular Junction**	1.35	*ACTG1*
**Caveolar-mediated Endocytosis Signaling**	1.31	*ACTG1*

Table S3. Diseases and functions with which the target proteins of baicalin are associated.

Category	p-value	Molecules (Gene names)
**Cancer**	5.41E-07	*ACTG1,ACTN4,HSP90AA1,HSP90AB1,HSPA1A/HSPA1B,NASP*
**Organismal Injury and Abnormalities**	5.41E-07	*ACTG1,ACTN4,HSP90AA1,HSP90AB1,HSPA1A/HSPA1B,NASP*
**Renal and Urological Disease**	5.41E-07	*HSP90AA1,HSP90AB1,HSPA1A/HSPA1B*
**Reproductive System Disease**	8.58E-07	*ACTG1,ACTN4,HSP90AA1,HSP90AB1,HSPA1A/HSPA1B,NASP*
**Connective Tissue Disorders**	1.24E-06	*HSP90AA1,HSP90AB1*
**Endocrine System Disorders**	1.24E-06	*HSP90AA1,HSP90AB1,NASP*
**Gastrointestinal Disease**	1.24E-06	*HSP90AA1,HSP90AB1*
**Hepatic System Disease**	1.24E-06	*HSP90AA1,HSP90AB1*
**Respiratory Disease**	1.24E-06	*HSP90AA1,HSP90AB1*
**Skeletal and Muscular Disorders**	1.24E-06	*HSP90AA1,HSP90AB1*
**Immunological Disease**	2.48E-06	*ACTG1,HSP90AA1,HSP90AB1*
**Developmental Disorder**	4.14E-06	*HSP90AA1,HSP90AB1*
**Dermatological Diseases and Conditions**	6.20E-06	*HSP90AA1,HSP90AB1*
**Tumor Morphology**	6.20E-06	*HSP90AA1,HSP90AB1*
**Hematological Disease**	1.16E-05	*ACTG1,HSP90AA1,HSP90AB1*
**Neurological Disease**	1.16E-05	*HSP90AA1,HSP90AB1*
**Auditory Disease**	2.27E-05	*HSP90AA1,HSP90AB1*
**Cellular Movement**	2.87E-05	*ACTG1,ACTN4,ATP5F1B,C1QBP,HSP90AA1,HSP90AB1,HSPA1A/HSPA1B,NPM1,YWHAQ,YWHAZ*
**Post-Translational Modification**	4.33E-05	*HSP90AA1,HSPA1A/HSPA1B*
**Protein Folding**	4.33E-05	*HSP90AA1,HSPA1A/HSPA1B*

**Cellular Compromise**	4.55E-05	*ACTN4,HSP90AA1,HSP90AB1,HSPA1A/HSPA1B,PRDX4*
**Inflammatory Response**	4.55E-05	*ACTN4,HSP90AA1,HSP90AB1,HSPA1A/HSPA1B,PRDX4*
**Infectious Diseases**	7.04E-05	*HSP90AA1,HSP90AB1*
**Ophthalmic Disease**	7.82E-05	*HSP90AA1,HSP90AB1*
